# Visible and UV photo-detection in ZnO nanostructured thin films via simple tuning of solution method

**DOI:** 10.1038/s41598-017-15125-x

**Published:** 2017-11-08

**Authors:** Richa Khokhra, Bandna Bharti, Heung-No Lee, Rajesh Kumar

**Affiliations:** 1 Jaypee University of Information Technology, Waknaghat, Solan, 173234 India; 2 Gwangju Institute of Science and Technology (GIST), 123 Cheomdangwagi-ro Buk-gu, Gwangju, 61500 South Korea

## Abstract

This study demonstrates significant visible light photo-detection capability of pristine ZnO nanostructure thin films possessing substantially high percentage of oxygen vacancies $({V}_{o}{\rm{s}})$ and zinc interstitials $(Z{n}_{i}{\rm{s}})$, introduced by simple tuning of economical solution method. The demonstrated visible light photo-detection capability, in addition to the inherent UV light detection ability of ZnO, shows great dependency of ${V}_{o}{\rm{s}}$ and $Z{n}_{i}{\rm{s}}$ with the nanostructure morphology. The dependency was evaluated by analyzing the presence/percentage of ${V}_{o}{\rm{s}}$ and $Z{n}_{i}{\rm{s}}$ using photoluminescence (PL) and X-ray photoelectron spectroscopy (XPS) measurements. Morphologies of ZnO viz. nanoparticles (NPs), nanosheets (NSs) and nanoflowers (NFs), as a result of tuning of synthesis method contended different concentrations of defects, demonstrated different photo-detection capabilities in the form of a thin film photodetector. The photo-detection capability was investigated under different light excitations (UV; 380~420 nm, white ; λ > 420 nm and green; 490~570 nm). The as fabricated NSs photodetector possessing comparatively intermediate percentage of ${V}_{o}{\rm{s}}$ ~ 47.7% and $Z{n}_{i}{\rm{s}}$ ~ 13.8% exhibited superior performance than that of NPs and NFs photodetectors, and ever reported photodetectors fabricated by using pristine ZnO nanostructures in thin film architecture. The adopted low cost and simplest approach makes the pristine ZnO-NSs applicable for wide-wavelength applications in optoelectronic devices.

## Introduction

Photodetectors have a wide range of applications in many important areas such as; space communication, air quality monitoring, flame monitoring, industrial quality control, optical imaging, optoelectronic circuits, military surveillances etc.^1^. Conventional photodetectors employ crystalline semiconductor materials such as; silicon, germanium, gallium arsenide etc. However, in these materials certain issues still need to be addressed; for instance, requirement of high temperature conditions for device fabrication, possibility of blurring, cross talk of optical signals between neighboring pixels^2^ and limited freedom in material design. To overcome these problems, studies on inorganic semiconductor nanostructures^3,4^ such as; ZnS, InSe, CdS, CdSe etc. and metal-oxide semiconductor^5^ such as; ZnO^6^, CeO_2_^7^,V_2_O_5_^3^ etc. could pave the way of fabricating a suitable photodetector. Nonetheless, these materials in nanostructure form provide a higher degree of freedom for material’s properties tuning as well as the reduced dimensionality of the active device^8,9^. However, as a key issue, these materials in their pristine form work only for ultra violet (UV) photo-detection applications, as allowed by their wide bandgap structure. Most of the studies correlate their wide bandgap with UV applications; however, in addition to the UV applications there are many areas that urgently require photodetector’s sensitivity for visible-light region, and thus there is a great need to achieve a wide spectral response of the proposed nanostructured semiconductor materials. In other words, the widening of photodetector’s spectral response (extended wavelength photo-detection) would enhance their application area. In this view, studies on the detection of visible spectrum by achieving a broadband photo-detection capability of nanostructured semiconductor materials, specifically metal-oxide semiconductors, have attracted a great attention in the last few years^7,10^.

The wide spectral applications of metal-oxide semiconductor materials require tuning of optical properties (bandgap) of semiconductor nanostructures; therefore, normally they are doped with metals^11^, non-metals^12^, combined with other materials/functional groups^13–15^, and formed as composites with another semiconductor materials^1,7,16^. However, it is noteworthy that most of these processes applied for tuning of optical properties, require complicated and expensive equipments, and a complex device structure to achieve visible-light detection response. Moreover, in these approaches, the requirement of high temperature and pressure conditions is an another issue. In this concern of application, when considering morphology, the one dimensional (1D) nanostructures owing to their large surface to volume ratio and Debye length (that influence electronic/optical properties and thus exhibiting superior photosensitivity) show better performance in the photo-detection applicaion^3,5^.

While talking about metal-oxide semiconducting materials, investigated for photo-detection applications, the ZnO is found most promising candidate due to its many peculiar properties such as; high efficiency, low cost, non-toxicity, stability, high temperature operation capability, and environmental compatibility. Looking to the superior photosensitivity of morphologically 1D nanostructure, the ZnO itself is also studied mostly in 1D form with various modifications such as; decorating with gold nanoparticles^17^ and CdS^18^, doping with Cu^19^, Mn^20^ and making heterostructures^21,22^ to show multi-spectral visible and UV light photo-detection capability. In principle, in the modifications, mid-gap electronic levels of dopant/s are introduced which generate charge carriers upon visible-light irradiation and thus make the material sensitive to visible light.

In a further and recent advancement^10^, undoped ZnO nanowires with a vertical alignment have been presented to exhibit an extended-range of visible light photo-detection capability upon annealing in hydrogen gas. The hydrogen annealing creates porosity on the surface of the nanowires that makes the assembled nanowire photodetector as a visible-light sensor by the phenomenon comprising antireflection, multiple scattering and defect state excitation induced mechanism. Similarly, the undoped ZnO structures have demonstrated visible-light activity upon vacuum deoxidation^23^, where oxygen energy levels are introduced in the energy band of ZnO. The introduction of oxygen levels in the band gap enables ZnO as an active material for visible and UV-light photo-detection. Despite of many efforts, in the case of undoped ZnO nanostructures, it is still a challenging issue to make a simple and economical photodetector that could use an easily fabricated nanostructure to work in broad spectrum region (ultraviolet and visible), except the lD nanostructures, and avoids the require sophisticated instrumentation. From the reported studies, it is ensured that for an undoped/pristine ZnO photodetector, it is only the multiple scattering or defects (${V}_{o}{\rm{s}}$,$Z{n}_{i}{\rm{s}}$ and antsites) that enables the visible-light/broadband response. Therefore, the tuning of morphology of undoped ZnO nanostructures, capable of broadband spectral-response through the combined effect of multiple scattering and formation of ${V}_{o}{\rm{s}}$ and $Z{n}_{i}{\rm{s}}$, synthesized by the simple solution method would be highly valuable for making an economical photodetector.

In this work, different morphologies of *undoped ZnO nanostructures*; NSs, NFs and NPs were formed simply by low temperature chemical route engineering. The ${V}_{o}{\rm{s}}$ and $Z{n}_{i}{\rm{s}}$ were introduced intentionally during the formation, so as to avoid the cost effective approaches^23^ which involve high temperature and vacuum conditions for inclusion of ${V}_{o}{\rm{s}}$ and $Z{n}_{i}{\rm{s}}$. Thus generated ${V}_{o}{\rm{s}}$ and $Z{n}_{i}{\rm{s}}$ levels in the energy band of ZnO exhibited significant photo-response in the visible as well as UV spectral region. The photo-response of these nanostructures was investigated in terms of generated photovoltages by wide range of spectral illumination i.e. λ ≈ 380~420 nm (UV), λ ≈ 490~560 nm (green), and λ > 420 nm (white light). We found that from the fabricated nanostructures, the NSs photodetector in a thin film form shows a faster rise and decay time both in the UV and visible spectrum region than that of the co-fabricated NPs and NFs photodetectors, and the ever reported sophisticated single ZnO nanowire based photodetector working only in UV region^24–26^. Based upon the observations, simply fabricated NSs, a low-cost photodetector could be a highly competent candidate for the applications requiring detection of wide range spectrum.

## Results and Discussion

### Morphological and X-ray analysis

**Figure 1:**
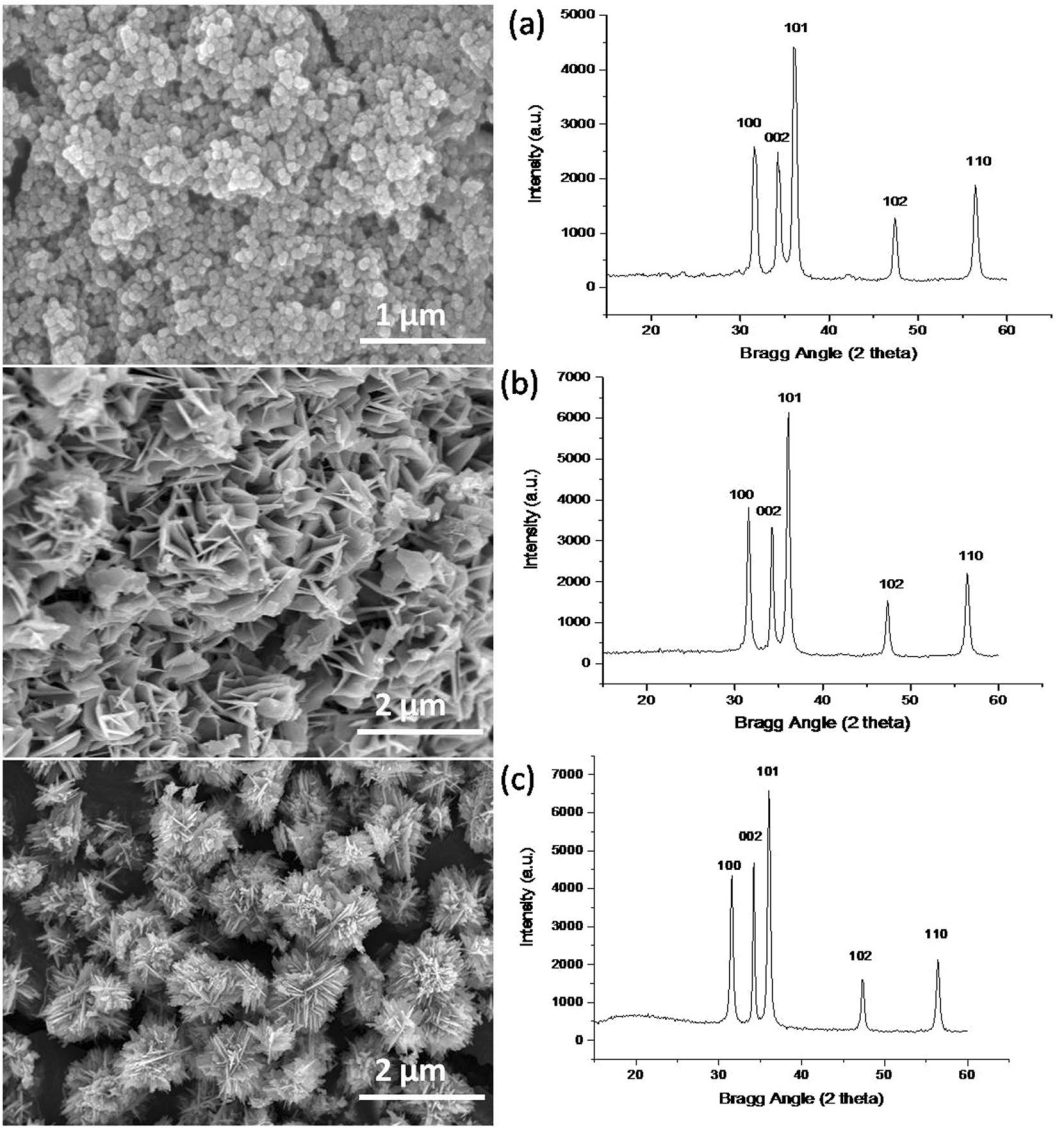
FE-SEM images and XRD results of ZnO nanostructures. (**a**) NPs formed in 15 minutes by using C_2_H_5_OH solvent, (**b**) ZnO-NSs formed in 4 hours using C_2_H_5_OH solvent and (**c**) ZnO-NFs formed in 4 hours using H_2_O solvent. In the right side, there are XRD plots indicating similar crystallographic structures for all the morphologies.

Surface morphology of the samples prepared by varying the synthesis parameters such as; variation in the concentration of precursor solutions, ratio, reaction time and solvent, was investigated using FE-SEM (Supplementary Figures S2 and S3 for C_2_H_5_OH medium and S4, S5 for H_2_O medium). The large number of synthesis reaction performed using the precursor ‘ZnCl_2_’ in solvent media C_2_H_5_OH and H_2_O resulted mainly in three types of nanostructures i.e. NPs (for 15 minutes of reaction time in both the reaction media), NSs and NFs that were formed as the only distinguishable forms of the product as shown in Fig. 1. The solvent media C_2_H_5_OH and H_2_O play a significant role in the different aggregations of initially formed nanoparticles that resulted in NSs and NFs after 4 hours of reaction time. The FE-SEM images show that an abrupt addition of ZnCl_2_ solution in alkali solution resulted preliminary in the formation of NPs (Fig. 1a) for both the solvent media, which then aggregated differently as NSs (Fig. 1b) and NFs (Fig. 1c), respectively. The XRD patterns are shown in the right of Fig. 1. There are five prominent diffraction peaks, in all the cases, at diffraction angles 2θ = 32.7°, 34.5°, 36.42°, 47.44°and 56.58°, which are indexed as lattice planes (100), (002), (101), (102), and (110) with the lattice constants (a = 0.325 nm and c = 0.5211 nm), corresponding to Wurtzite crystal structure of ZnO. The size of nanocrystals estimated for maximum intensity peak, using Debye-Scherrer formula D = 0.9λ/*β*cos*θ* is 17.41 nm, 21.57 nm and 23.29 nm for NP, NS and NF, respectively; with an average crystallite sized 20.16 nm, 24.44 nm and 20.44 nm for NP, NS and NF, respectively, indicates a successive growth mechanism of nanostructures evolving from the nanoparticles.

**Figure 2:**
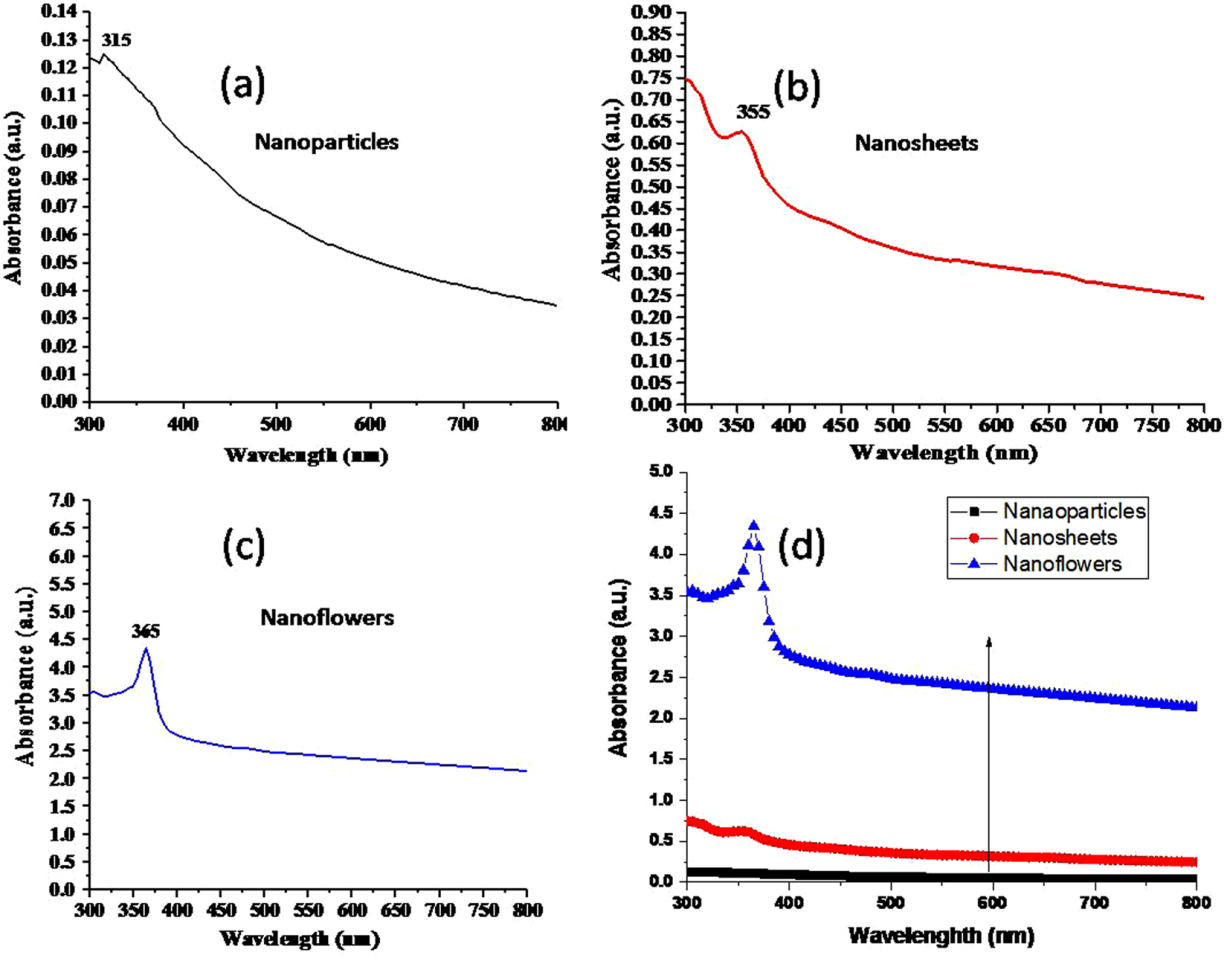
UV-Vis spectroscopy absorbance spectra. (**a**) NPs having excitonic peak at 315 nm and extended absorbance in the visible spectrum region, (**b**) NSs having excitonic peak at 355 nm and showing extended visible region, (**c**) NFs having excitonic peak at 365 nm with comparatively higher absorbance, and (**d**) shows increasing absorbance from NPs to NSs and then to NFs.

Optical absorbance of the samples obtained by UV-Visible spectroscopy is shown in Fig. 2. These absorption spectra appear to be extended from UV to visible-region, with a sharp UV excitonic peak at wavelength, 315 nm for NPs (Fig. 2a), 355 nm for NSs (Fig. 2b) and 365 nm for NFs (Fig. 2c). The presence of excitonic peak in UV region along with an extended absorption region reveals their UV as well as visible-light activity^10^. The shift in the excitonic peak from NPs (315 nm) to NSs (355 nm) and then to NFs (365 nm), corresponds to a red shift in the spectrum. With the shift in the excitonic peaks, the overall absorbance also increases from NPs to NSs and then to NFs (Fig. 2d). This result is analogous to the observations of enhanced absorbance in the porosity induced antireflections leading to visible-light photo-activity^10^. A rough estimation of the porosity order as NFs > NSs > NPs, can be made from the FE-SEM images of ZnO films (Fig. 1). Among these nanostructures, the NFs are expected to have comparatively more multiple reflections of light rays once they enter the film, and these large multiple reflections may lead to higher absorption^10^ in the NFs film in comparison with NSs and NPs films as shown in Fig. 2d.

### PL and XPS studies

**Figure 3:**
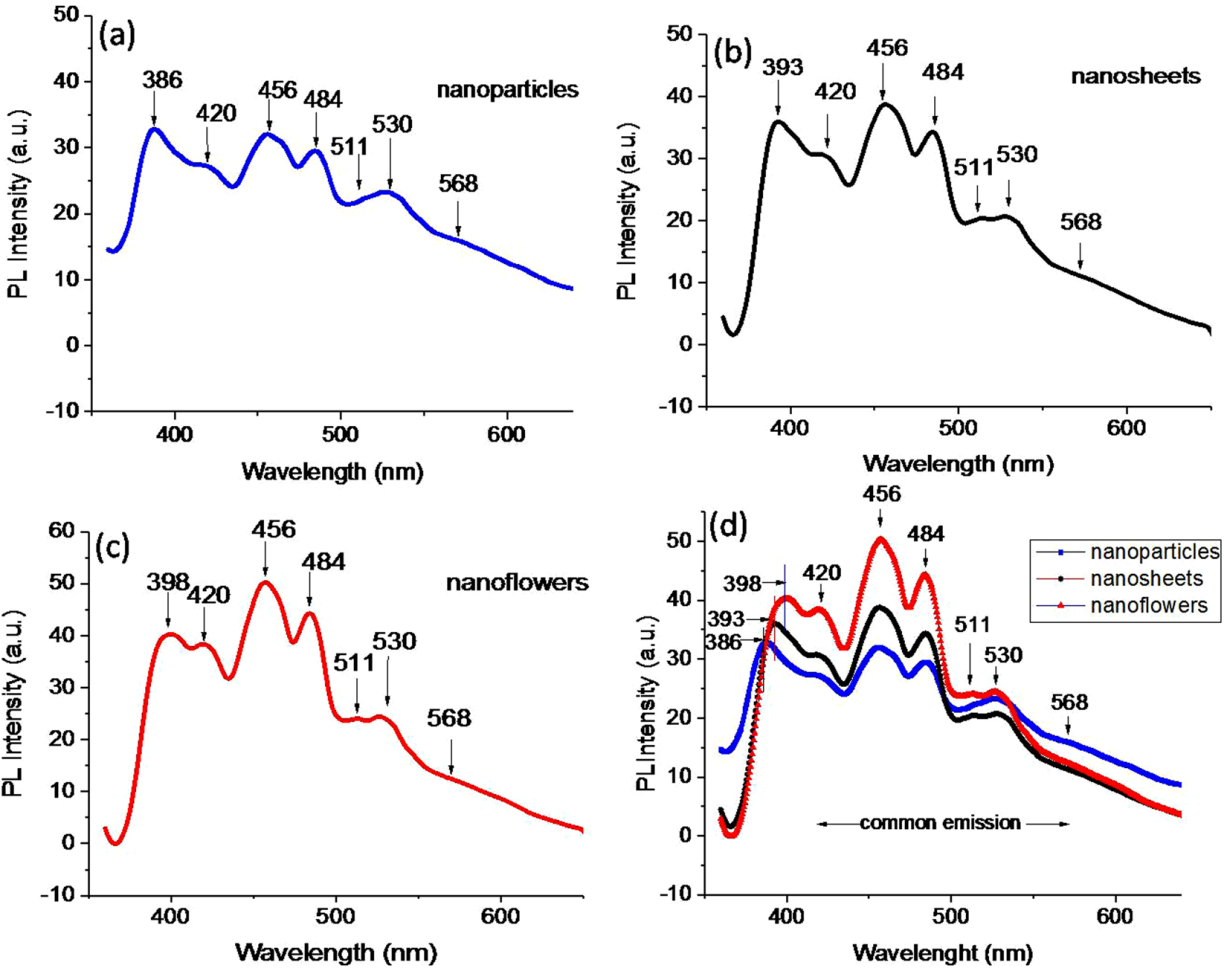
Photoluminescence spectra of nanostructures. (**a)**NPs show violet emissions at 386 nm, **(b)** NSs show violet emissions at 393 nm and **(c)** NFs show violet emission at 398 nm. Blue emissions at wavelengths positions 420, 456 and 848 nm and green emissions at wavelengths 511, 530 and low intensity peak at 568 nm are common in all nanostructures.

Emission/absorption in the UV and visible-region by ZnO nanostructures were investigated via analyzing defects states (vacancies, interstitials and antisite) in the studies conducted by PL and XPS experiments. Figure 3 shows a room temperature PL spectrum of NPs, NSs and NFs nanostructures. For excitation energy ~3.36 eV (corresponding to wavelength ~370 nm, equivalent to the typical band gap of ZnO), the PL spectra of NPs, NSs and NFs samples show near-band emission; respectively, at 386 nm (3.21 eV) (Fig. 3a), 393 nm (3.15 eV) (Fig. 3b) and 396 nm (3.12 eV) (Fig. 3c) which is attributed to the transitions from excitonic levels and/or zinc interstitials $(Z{n}_{i})$ to the conduction band (*CB*)^27^ and is analogous to the previous studies^28,29^. Along with these near-band emissions, all three samples exhibit visible emissions in the spectrum region 420–569 nm (Fig. 3d) consisting distinct peaks at 420 nm (2.95 eV),456 nm (2.71 eV),484 nm (2.56 eV), 511 nm (2.42 eV),530 nm (2.34 eV) and 568 nm (2.43 eV). Basically, transition from *VB* to *CB* and from *VB* to shallow levels occurs upon photoexcitation in the PL, which then give the subsequent transitions; *CB* → deep levels, shallow levels → *VB*, shallow level → deep levels and hole capture at deep levels gives violet, blue and green emissions according to energy levels difference. Since the exciting energy (3.36 eV) is equivalent to ZnO energy gap; therefore, the electrons excited from the valance band (*VB*) can jump to the *CB* as well as shallow defect levels.

Generally, oxygen vacancies (${V}_{o}^{* }$, $\,{V}_{o}^{+}{\rm{and}}\,{V}_{o}^{++}$) are considered as green emission centers, where ${V}_{o}^{* }$ is neutralized oxygen vacancy that lies 0.86 eV below the *CB*, and the ${V}_{o}^{++}$ a double ionized vacancy lies ~2.18 eV below the *CB*. The single ionized oxygen vacancy $\,{V}_{o}^{+}$ is reported to have two energy locations; 0.9 and 2.47 eV above the *VB* in the band gap^30,31^. Besides the oxygen vacancies, oxygen antisite $({O}_{Zn})$ located 2.33 eV below the *CB* do corresponds to green emission of shorter wavelength^30^. Other defects such as; zinc interstitials $Z{n}_{i}{\rm{s}}$, natural $(Z{n}_{i})$, singly and doubly ionized interstitials ($Z{n}_{i}^{+}$, $Z{n}_{i}^{++}$) are responsible for blue emission^27,32,33^. $\,Z{n}_{i}$ being located 0.22 eV below the *CB* edge^34^ gives violet emission around 390 nm in the PL. However, some studies show more deeper location of $\,Z{n}_{i}$ (~0.37 eV below *CB*) to explain violet-blue emission^27^. Other two Zn defects; $Z{n}_{i}^{+}$ and $Z{n}_{i}^{++}$ lie 0.56 and 0.63 eV below the *CB* minima^27^, respectively; whereby the transitions to zinc vacancy $({V}_{Zn})$ and/or *VB* results in blue emissions of higher wavelengths^35^. Except $Z{n}_{i}{\rm{s}}$ and ${V}_{Zn},\,$ the oxygen interstitial $({O}_{i})$ generally located in the band gap at position 0.4 eV above the *VB*, also participates in the blue emission^35,36^.

**Figure 4:**
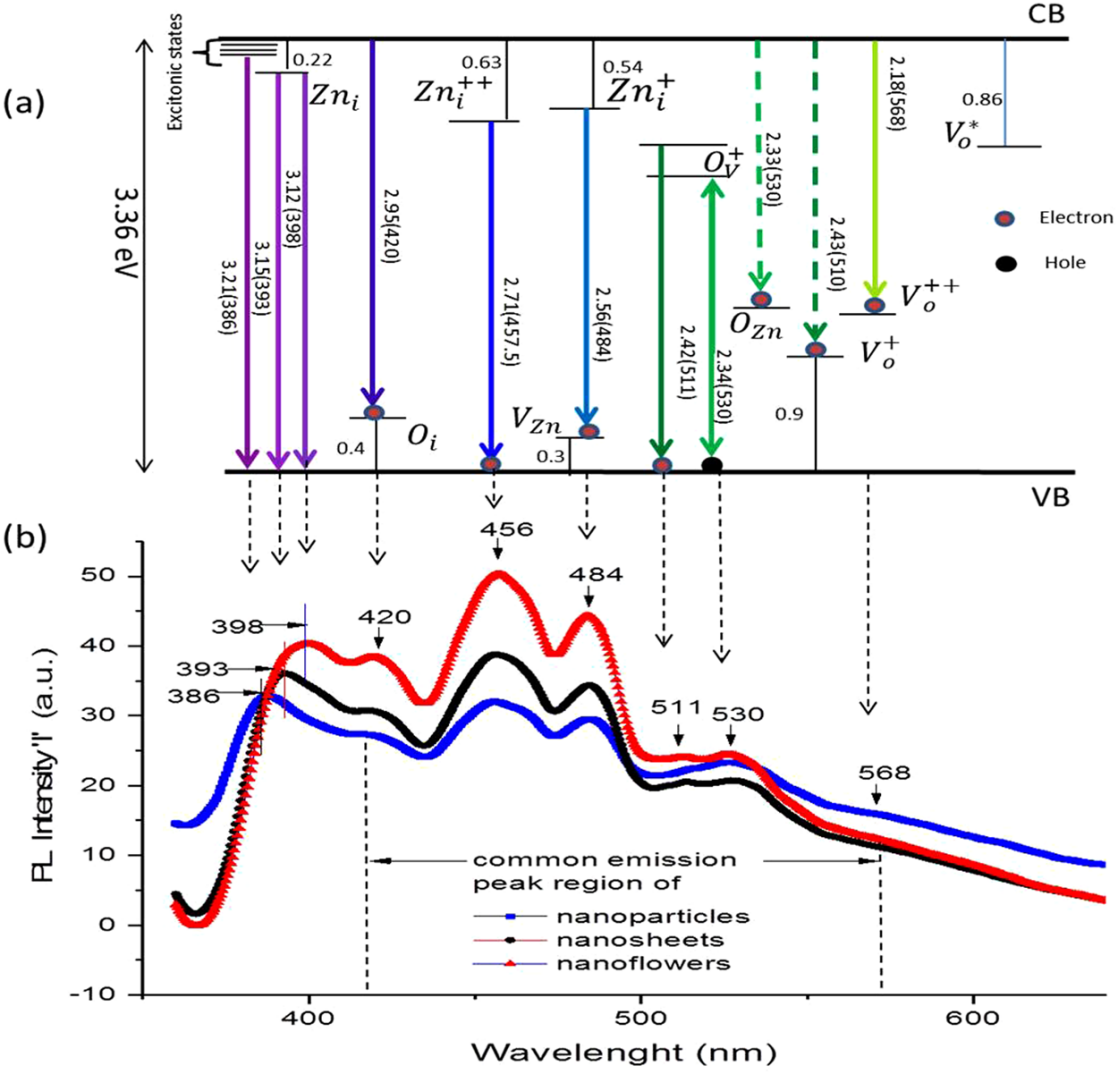
Emission scheme with PL spectra. (**a**) Schematic representation of emission scheme in the prepared samples of NPs, NSs and NFs nanostructures, (**b**) PL emission showing different peak position in violet region 386–398 nm, and common emission peaks in the visible region 420–568 nm in all samples.

PL spectra in Fig. 3 show co-existence of violet emission peak in the range 386–398 nm (corresponding to excitonic emissions) and visible emission peak in the range 420–568 nm. All three types of nanostructures; NPs (3a), NSs (3b) and NFs (3c) show common visible light emission peaks at 420 nm (2.95 eV), 456 nm (2.71 eV), 484 nm (2.56 eV), 511 nm (2.42 eV), 530 nm (2.34 eV) and a low intensity peak at 568 nm (2.43 eV), whereas UV region emission peaks are located at different wavelengths. The UV emission peak shows a red shift in a order NPs → NSs → NFs as can be seen in Fig. 3d. This red shift in PL is analogues to the observed red shift in UV-Vis spectrum (Fig. 2), indicating slightly different excitonic energy levels in the band gap of synthesized nanostructures. As reported earlier ZnO possesses stable excitonic states just below its *CB* ^33^ minima, whereby transition to *VB* gives near band violet emissions. Thus in our case, the observed peak in PL of NPs at position 386 nm (3.21 eV) can be assigned to the transition from excitonic states to its *VB*. In other words, the decay of self-trapped exciton to the *CB,* causes near band violet emission as shown in Fig. 4. Similarly, the UV emissions in NSs and NFs at 393 nm (3.15 eV) and 398 nm (3.12 eV) are close to the electronic transition from a slightly lower energy excitonic state or Zn interstitial $\,Z{n}_{i}$ (lying ~0.22 eV below the conduction band) to the *VB*. The possible transition scheme, corresponds to all the peaks in the PL shown in Fig. 4b, is given in Fig. 4a.

In the visible region, the observed blue emission peak at 420 nm (2.95 eV) corresponds to the transition from $\,Z{n}_{i}\,$ to *VB*, considering that $\,Z{n}_{i}\,$ lies ~0.41 eV deeper to the *CB* edge, this is alike to the previous reports^24^, wherein energetic location of $\,Z{n}_{i}\,$ is considered 0.38 eV deeper in the energy band. The another possibility is the transition *CB* → oxygen interstitial ${O}_{i}$ (located 0.4 eV above VB as proposed earlier^35^) that also gives blue emission at 420 nm. In fact, the second one is rather in a good agreement as the energy level difference (2.96 eV) between CB edge and ${O}_{i}\,$ is close to the obtained emission energy (2.95 eV). Nonetheless, the excitation energy (3.36 eV) is quite enough to pump the electrons from *VB* to *CB* that make a transition CB → ${O}_{i}$ and emit blue radiation of 420 nm, indicating the latter transition more plausible. The another blue emission at ~456 nm (2.71 eV) is assigned to the transition from extended $\,Z{n}_{i}\,\,$ states → *VB*. The extended $\,Z{n}_{i}$ states are generally; localized $\,Z{n}_{i}$ states,$\,Z{n}_{i}^{++}$ and complex defects, whose energetic locations deep in the band gap depend upon the fabrication methods^27,35^. In our case, the blue emission (2.71 eV) is in close agreement to the energy of transition $Z{n}_{i}^{++}$ → *CB* (2.73 eV), indicating the presence of $\,Z{n}_{i}^{++}$ states in the band gap. Third blue emission peak located at 484 nm (2.56 eV) corresponds to the transition from $Z{n}_{i}^{+}$ → ${V}_{Zn}$, as the energy difference between these levels (2.52 eV) is in close agreement to the observed emission energy.

Green emissions falling in the spectral region 511–568 nm, possesses three emissions peaks at 511 nm (2.42 eV), 530 nm (2.34 eV) and 568 nm (2.18 eV). When the electrons from *CB*, recombine with doubly ionized ${V}_{o}^{++}\,$ located at an energy level 1.12 eV above the *VB*, generate green emission of wavelength 568 nm^37^. The second green emissions peak centered at 530 nm may come from the transition $\,{V}_{o}^{+\,}$ → *VB* or *CB* → ${O}_{Zn}$. The first transition ($\,{V}_{o}^{+\,}$ → *VB*) occurs due to the formation of unstable ${V}_{o}^{+}$ state of ${V}_{o}^{+}$ by capturing electrons form *CB* ^38^. This unstable state, when recombine with photoexcited hole in the *VB*, would generate green emission around 530 nm^39^. The second transition *CB* → ${O}_{Zn}$ also has a strong possibility as the exciting energy is enough to pump the electrons to *CB* that after falling to ${O}_{Zn}$ will give rise a green emission at 530 nm. Coming back to the ${V}_{o}^{+}$ states, in the energy band, there may be occurrence of complex ${V}_{o}^{+}\,$ states along with isolated ${V}_{o}^{+}\,$ centers. The complex states lying deeper in the band gap also give a possible explanation of the green emission around 530 nm, whereas the isolated ${V}_{o}^{+}\,$ states suitably explain the emission around 511 nm^38^ through the transition ${V}_{o}^{+}$ → *VB*. As mentioned previously, two possible energetic locations of isolated ${V}_{o}^{+}$ states are estimated theoretically at 0.9 and ~ 2.47 eV above the *VB*. The transition ${V}_{o}^{+}$ → *VB* (corresponding to 2.47 eV energy level position of $\,{V}_{o}^{+}$) gives 511 nm emission as shown in the scheme of Fig. 4a, and the transition *CB* → ${V}_{o}^{+}$ (corresponding to 0.9 eV energy level position of ${V}_{o}^{+}$) will give 510 nm emission as shown in the scheme of Fig. 4a by dashed line. Both of the transitions have equal possibility to generate emission around 510 nm in the PL. All these observations in the PL, indicate that all of the samples possess defects states such as; ${V}_{o}{\rm{s}}$, $\,Z{n}_{i}$, ${O}_{i}$, ${V}_{Zn}$ and ${O}_{Zn}$.

**Figure 5:**
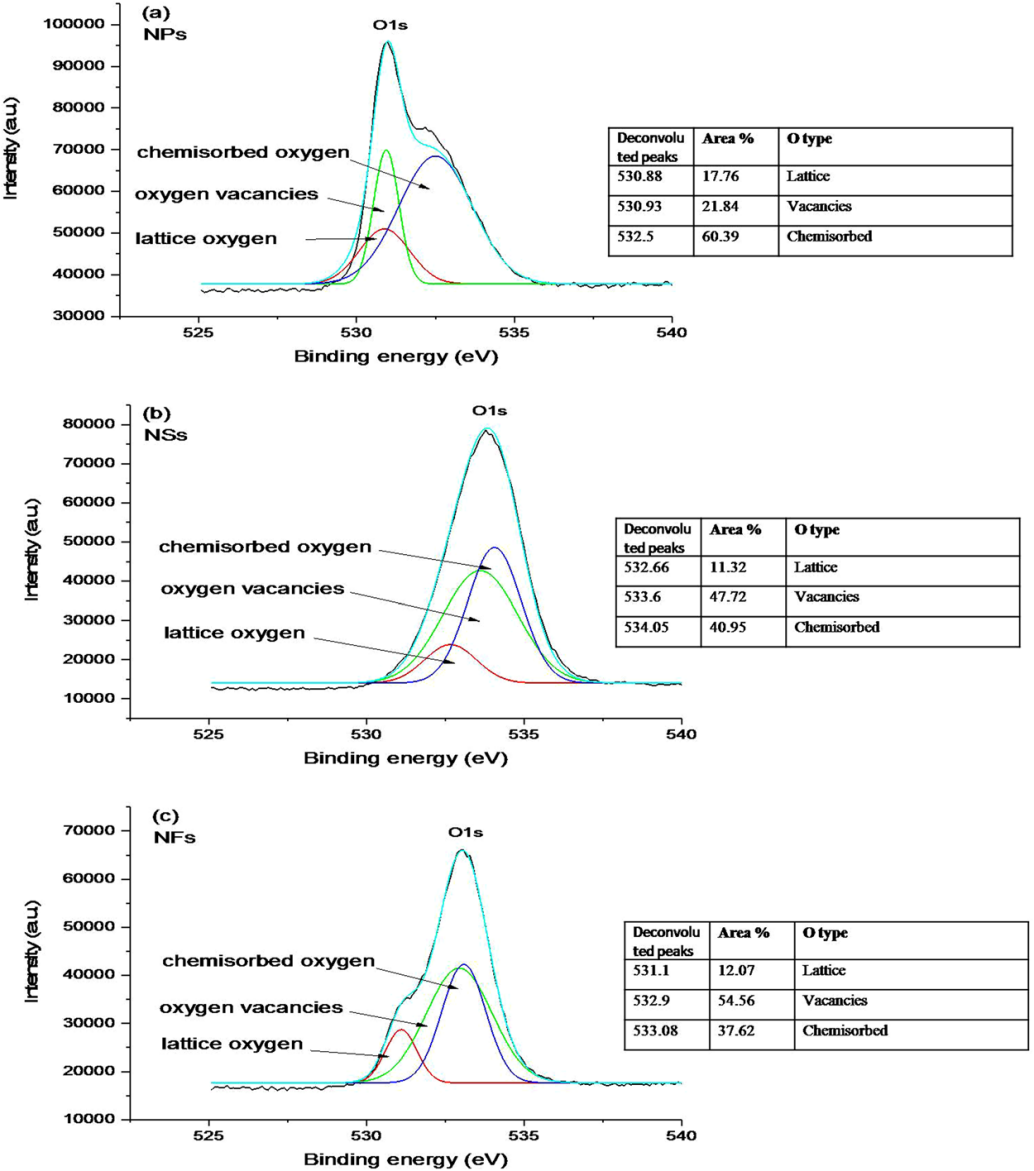
High-resolution XPS spectra for O1s. (**a)** ZnO-NPs different percentages of lattice oxygen, oxygen vacancies and chemisorbed oxygen, **(b)** ZnO-NSs and **(c)** ZnO-NFs show variations in the peak positions, and constituent percentages are given in respective tables.

XPS studies were performed to get information about chemical bonding and defects states present in the ZnO samples. Figure S6(a–c) shows XPS survey spectra recorded at room temperature for NPs, NSs and NFs samples. The overview of survey spectra reveals the presence of O1s and Zn2p (Zn2p_3/2_ and Zn2p_1/2_) peaks in all the samples. In order to further examination, the high-resolution peaks were deconvoluted in satellite components at different binding energies. Figure 5(a–c) illustrates high-resolution XPS spectra for all the samples corresponding to O1s core level. These spectra are fitted with three Gaussian peaks. In the NPs case (Fig. 5a), deconvoluted peaks are located at binding energies 530.8, 530.93 and 532.5 eV. Here, the lower binding energy peak is attributed to lattice oxygen (${O}_{L}$) which contributes to the perfect hexagonal structure of ZnO lattice, the presence of middle peak at binding energy 530.93 eV is ascribed to vacancies (${V}_{o}{\rm{s}}$)^40^ in ZnO lattice. The observation of ${V}_{o}{\rm{s}}$ supports the presence of green emission line in PL. The higher binding energy peak at 532.5 eV corresponds to chemisorbed oxygen (${{\rm{OH}}}^{-},$ −CO_3_, adsorbed H_2_O, and O_2_ (O_C_))^41,42^. Similarly, the peaks in high-resolution deconvoluted XPS spectrum of NSs and NFs, when analyzed do correspond to $\,{O}_{L}$, ${V}_{o}{\rm{s}}$ and chemisorbed oxygen. However, there is shift in the binding energy values of the corresponding deconvoluted peaks for NSs and NFs with respect to that of NPs, which is ascribed to the difference in their morphologies and synthesis approaches^43,44^. Further, the change in the percentage of oxygen content related to each deconvoluted peak was estimated by the change in percentage area of the peaks. From the calculations of percentage area, we found that the lattice oxygen in NPs is about 17%, whereas in case of NSs and NFs it reduces to ~ 12%. At the same time, the percentage area of oxygen vacancies (${V}_{o}^{\ast }$, $\,{V}_{o}^{+}\,{\rm{and}}\,{V}_{o}^{++}$)^45,46^, increases from 21.8% (in NPs) to 47.7% (in NSs) and 54.5% (in NFs), and that of the chemisorbed oxygen decreases from NPs (60.3%) to NSs (40.9%) and then to NFs (37.6%).

**Figure 6:**
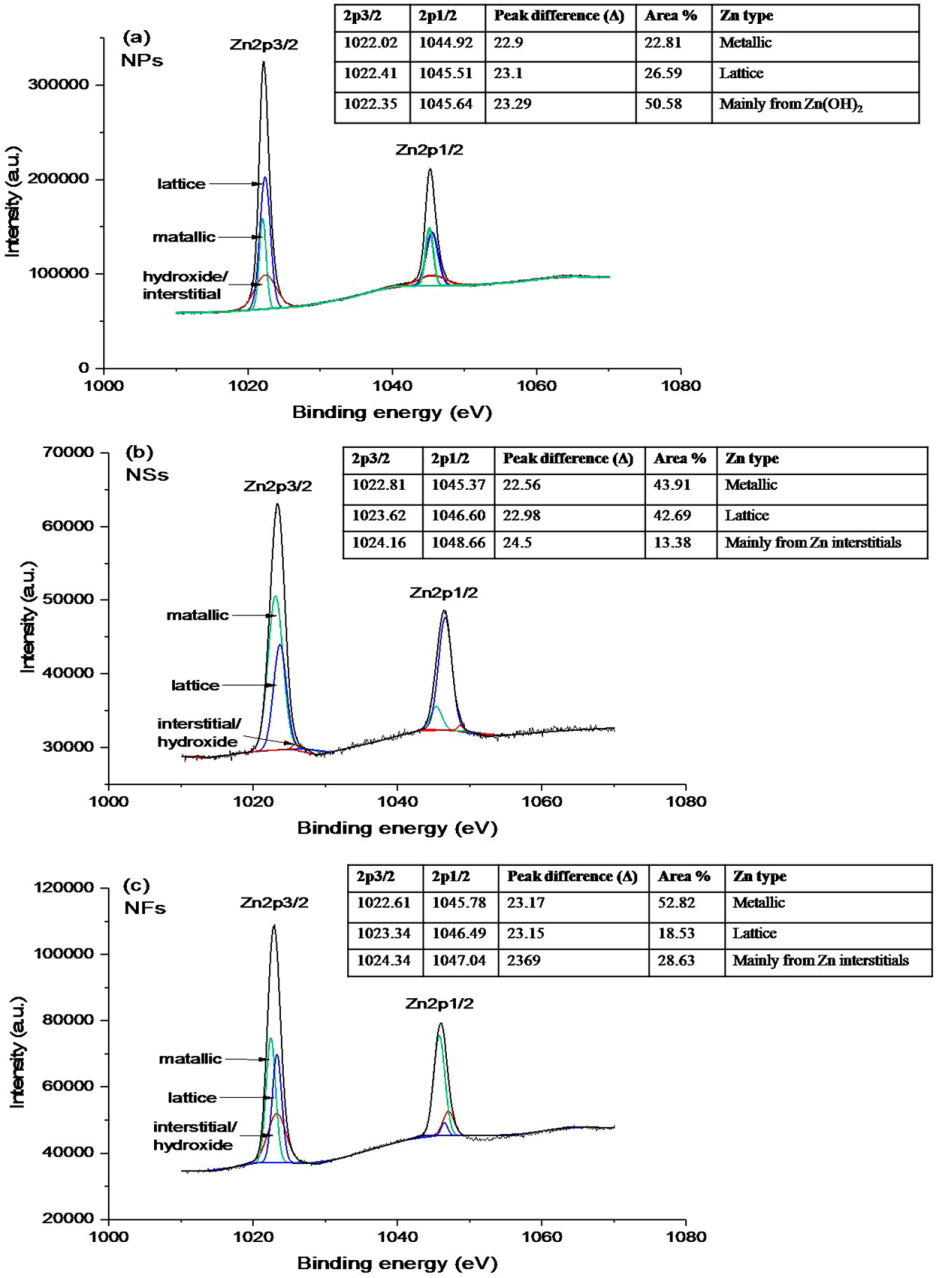
High-resolution XPS spectra of Zn2p. (**a)** ZnO-NPs possessing dominating Zn peak at higher energy side is due to the hydroxide form as mentioned in table, **(b)** ZnO-NSs and (c) ZnO-NFs spectra have dominating interstitial Zn peak at higher energy. The position of peaks and their area percentages are mentioned in the corresponding tables.

Figure 6(a–c) represents high-resolution XPS spectra for Zn peaks in the NPs, NSs and NFs samples, respectively. In these spectra, the energy separation between two Zn components; Zn2p_3/2_ and Zn2p_1/2_ for NPs is 23.11 eV, NSs is 23.09 eV and NFs is 23.13 eV, which are in agreement with the reported values of ZnO^47,48^. The difference of binding energies of these deconvoluted peaks for NPs, NSs and NFs (given in Table S2) is ascribed to the difference in their morphologies and synthesis approaches^43,44^. The peak Zn2p_3/2_ in NPs sample is deconvoluted in three peaks at binding energies 1022.02 eV (lower side), 1022.41 eV (middle) and 1022.35 eV (higher), also the peak Zn2P_1/2_ is deconvoluted in three peaks at binding energies 1044.92 eV (lower), 1045.51 eV (middle) and 1045.64 eV (higher) as shown in Fig. 6a. The middle peaks (1022.41 and 1045.51 eV) have an energy difference of 23.1 eV, which is in good agreement with the spin-orbit splitting value of divalent Zn bounded in ZnO structure^49,50^, and thus suggesting that the middle peak corresponds to lattice Zn. The lower energy peaks centered at 1022.02 and 1044.92 eV correspond to metallic Zn in the sample^51^. And, third peaks centered at higher energies 1022.35 and 1045.64 eV, correspond to +2 oxidation state of Zn due to the presence of Zn(OH)_2_ or/and ionized $Z{n}_{i}$ interstitials. It is found^37,52^ that the binding energy location of satellite peak of 2p_3/2_ lying between 1022.70 and 1021.80 eV corresponds to Zn(OH)_2_, and therefore the +2 oxidation state is due to the presence of Zn(OH)_2_. Whereas in our case for NPs sample, the satellite peak 2p_3/2_ (at 1022.35 eV) lies between the referred energy limits, which suggests the presence of Zn(OH)_2_ or +2 oxidation state by hydroxide form of Zn. This can be further correlated with the corresponding peak in the O1s spectrum of NPs (shown in Fig. 6a), which is assigned as chemisorbed oxygen (generally in OH^−^ and/or water molecules). In the O1s spectrum, the obtained higher percentage (60.39%) of chemisorbed oxygen can be assigned to ${{\rm{OH}}}^{-}$ group as confirmed form Zn2p_3/2_ spectrum; however, there should co-exist a small percentage of interstitial Zn as well, as to produce emission in the corresponding PL spectrum (as the PL of nanoparticles shows the presence of interstitials). Moreover, the lower PL emission intensity of NPs than that of NSs and NFs indicates comparatively smaller interstitial percentage in NPs. Next, in case of NSs, each of the peaks Zn2p_3/2_ and Zn2P_1/2_ (Fig. 6b) is deconvoluted in three satellite peaks at energies 1022.81, 1023.62, 1024.16 eV and 1045.37, 1046.6, 1048.66 eV, respectively. The middle peaks centered at binding energies 1023.62 eV and 1046.6 eV have energy difference of 22.98 (approximately 23.0 eV), that again corresponds to lattice Zn with two valance state in ZnO. The satellite peak at lower energy side is assigned to metallic Zn alike to that of the NPs sample. However, in this sample the higher energy satellite peaks 1024.16 and 1048.66 eV located at significantly higher energies do not correspond to Zn(OH)_2_; instead it indicates that the Zn atom is surrounded by more than one oxygen atoms and is occupied at interstitial positions^37^. These interstitial Zn could be neutral $\,Z{n}_{i}$, extended states of $\,Z{n}_{i};$ single and double ionized zinc interstitials ($Z{n}_{i}^{+}$ and $Z{n}_{i}^{++}$). Similarly, in the samples of NFs, the Zn2p spectrum is deconvoluted in three peaks as shown in Fig. 6c. Here also, the higher energy satellite peaks are located at much higher energies as 1024.34 and 1074.04 eV should correspond to the interstitial Zn similar to that of NSs sample. However, the area percentage in NFs is more than that of NSs, suggesting a higher content of interstitials in NFs, which is in good agreement with PL observation (Fig. 4b) showing higher intensity, revealing the higher Zn interstitials in NFs sample.

### Performance of ZnO photodetectors

**Figure 7:**
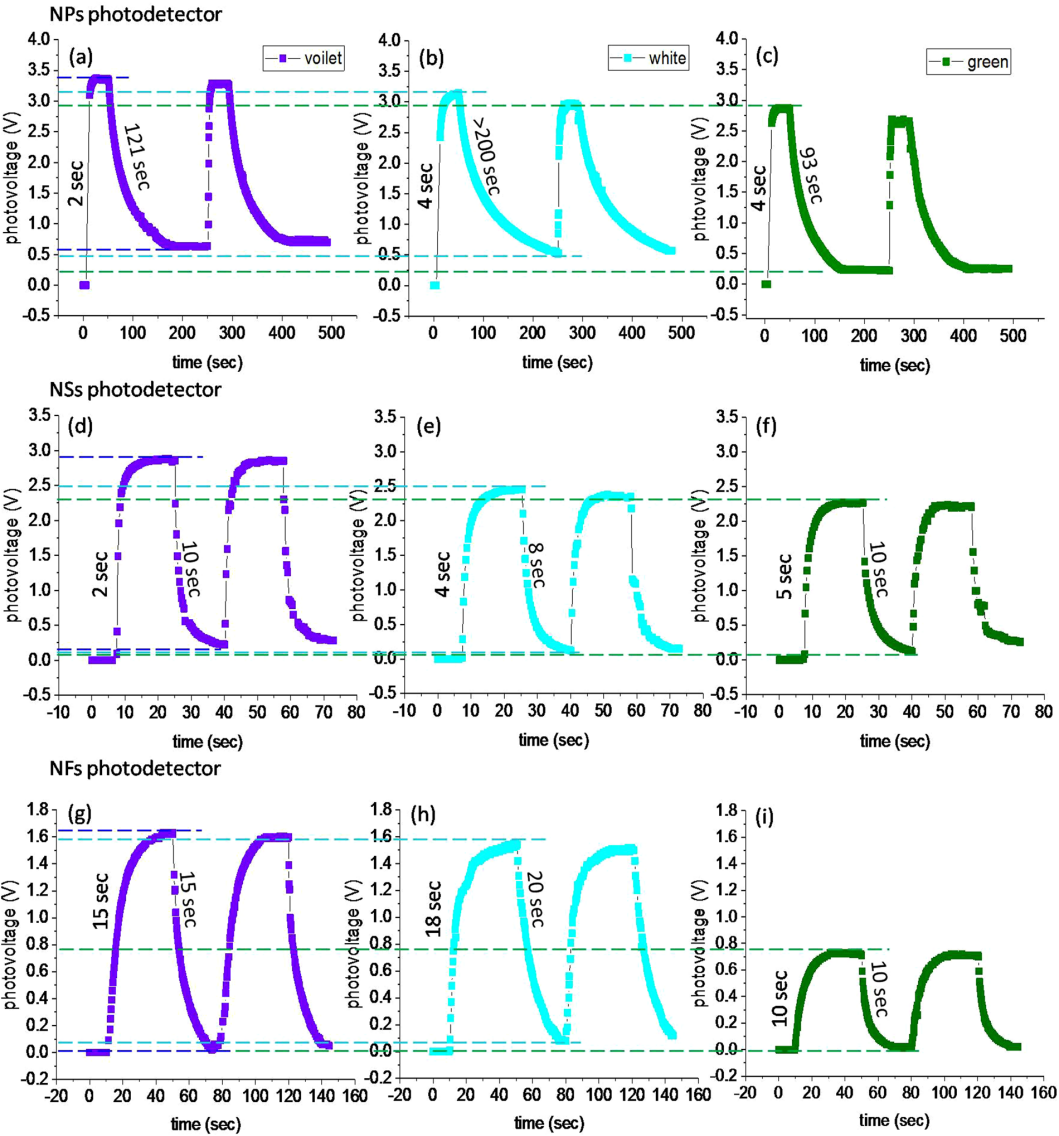
Response of nanostructured thin film photodetectors. (**a**–**c)** NPs photodetector, **(d**–**f)** NSs photodetector and **(g**–**i)** NSs photodetector for UV, white and green illuminations, dashed lines are plotted for clear observation of photo-generated ON state saturation voltages and OFF state dark voltages by different illuminations. In each photodetector, the illumination time is taken as short as required to avoid the heating effects. The applied bias voltage in all three cases is 5 V.

To study the performance of ZnO photodetectors, photo-response was measured in terms of photovoltage by applying a bias voltage ‘*V*_*b*_ = 5 V’ as shown in Figure S1. Figure 7 shows photovoltage versus time plots for all the three types of nanostructures; NPs (Fig. 7a), NSs (Fig. 7b) and NFs (Fig. 7c) thin film photodetectors under the illuminations; violet, white and green. In the photovoltage measurements, rise time is the time required in 90% rise of photovoltage form its initial value (after switching ON the illumination), and fall time is the time taken in the falling of maximum photovoltage to 10% (after switching ‘OFF’ the illumination)^53^. For each photodetector, the period of ON and OFF time was taken different by considering their response time as different, and also to eliminate the heating effects on the sample surface^39,54^. In the experiments, the ON and OFF time was controlled using a camera shutter. The photo-response of all ZnO nanostructure photodetectors (in the region ultraviolet to visible) is given in Table 1.

**Table 1:** Shows comparison of the performance of ZnO nanostructures-based photodetectors under different spectral illumination using 5 V bias voltage.

Structures/photodetectors	Saturation photovoltage for each illumination (V)	Growth time for each illumination (sec)	Decay time for each illumination (sec)	Surface area (m^2^/gm)
NPs	Violet: 3.35	UV ~ 2	Violet~121	20.5
	White: 3.12	White ~ 4	White > 200	
	Green: 2.85	Green ~ 4	Green ~ 93	
NSs	Violet: 2.87	UV ~ 2	Violet ~ 10	11.8
	White: 2.45	White ~ 4	White ~ 8	
	Green: 2.27	Green ~ 5	Green ~ 10	
NFs	Violet: 1.62	UV ~ 15	Violet ~ 15	10.0
	White: 1.53	White ~ 18	White ~ 20	
	Green: 0.72	Green ~ 10	Green ~ 10	

The mechanism of photodetector response with light illumination can be explained by desorption and adsorption of O_2_ and/or H_2_O on its surface^26,55–58^. In a dark condition, O_2_ and H_2_O molecules present in the air get adsorbed on ZnO surface, which by capturing electrons from the conduction band of ZnO becomes negatively charged ions (chemisorbed) that results in the increased depletion barrier height. When the illumination is turned on, the chemisorbed O_2_ gets H_2_O is/are desorbed by two ways; (i) capturing a photo-generated hole and/or (ii) direct photo-excitation of captured electron to the conduction band of ZnO^24^. The desorption mechanism occurs in accordance to the energy level of illuminating radiation (above-band or below-band energy). While using above-band illumination ‘UV radiation’, electron and hole are generated directly since the illumination energy is higher than the band gap of ZnO. Thus the produced photo-holes migrate to the chemisorbed ions sites, and release the electron by neutralizing the ions. The desorption of ions decreases barrier height and releases electrons in the conduction band of ZnO. These released electrons along with the photo-electrons would enhance the concentration of carriers in the *CB* of ZnO, and thus give rise to its photoconductivity. In case of below-band illumination, desorption of O_2_/H_2_O occurs by direct photo-excitation of the captured electrons to the conduction band of ZnO that also increases photoconductivity. In the present study, we used above-band (UV) as well as below-band (white and green) illuminations which resulted in the generation of photovoltage. Thus the observed photovoltage might involve both of the desorption mechanisms explained above, according to the illumination conditions.

First of all, we compare photo-response curves corresponding with different illuminations in the NPs photodetector. In the NPs photodetector, the photovoltage initially at ~0 V (dark voltage) reaches the saturation voltage 3.35 V within 2 sec upon UV exposure (Fig. 7a), and at 3.12 V and 2.85 V within 4 sec after white and green illuminations (Fig. 7(b,c)), respectively. Here, the illumination ON and OFF time were taken as 50 sec and 200 sec, respectively. While looking at the photo-response curve of NPs photodetector, the rise time corresponding to different illuminations have small variations, whereas their decay time have large difference, and are much longer in comparison with the corresponding rise time as 93 sec for green, 121 sec for UV and greater than 200 sec for white illumination. After turning OFF the illuminations, the photodetector does not achieve its original dark voltage; instead it remains at a minimum voltage such as; 0.635 V for UV, 0.241 V for green illuminations, whereas for white illumination, it remains unsaturated even after 200 sec as can be seen in Fig. 7a.

The observed difference in the photovoltage rise time with UV, white and green illuminations can be understood by the difference in their illumination energies. UV illumination being an above-band energy, generates carriers both by desorption of adsorbed ions as well as direct excitation of electrons from *VB* to *CB*, whereas in case of white and green illuminations the charge carriers are generated only by desorption of the adsorbed ions. Thus for UV illumination, the reduction in depletion region height should be larger, enhancing charge carriers mobility^59^, and hence the fast rise in the photovoltage (Table 1). In the response curves, the ON state saturation voltage is also a reflection of different illumination energies. Based upon the energy levels of the illuminations, different concentrations of charge carriers are generated that give rise to the different values of saturation photovoltages as shown in Fig. 7. The decay of photovoltage after turning OFF the illuminations is due to re-adsorption of O_2_/H_2_O. The re-adsorption/decay curve shows two regions, fast decaying and slows decay regions. The fast decay is due to an instantaneous electron capture by adsorbed chemisorbed O_2_/H_2_O on the surface ${V}_{o}{\rm{s}}$, and slower decay is due to the adsorption of O_2_/H_2_O deep into the surface between nano-crystallites^60^. The latter involves rate-limited diffusion and rearrangement of O_2_/H_2_O in a closed packed structure for adsorption on the surface that makes the process slower^60^. Just after turning OFF the illuminations, the decrease in photovoltage is faster and almost similar for all three illuminations (UV, white and green), which successively becomes slower on the latter stage and acquires different decreasing rate for each illumination.

A model governing the rate-limited diffusion controlled adsorption process can be represented as^60^;

1 $$\frac{dNi(t)}{dt}=\frac{Ns-Ni(t)}{\tau }$$

where N*i* represents density of charged ions (after capturing electrons), N*s* is saturation density of ionized species which prevents further ionization and τ is adsorption rate.

**Figure 8:**
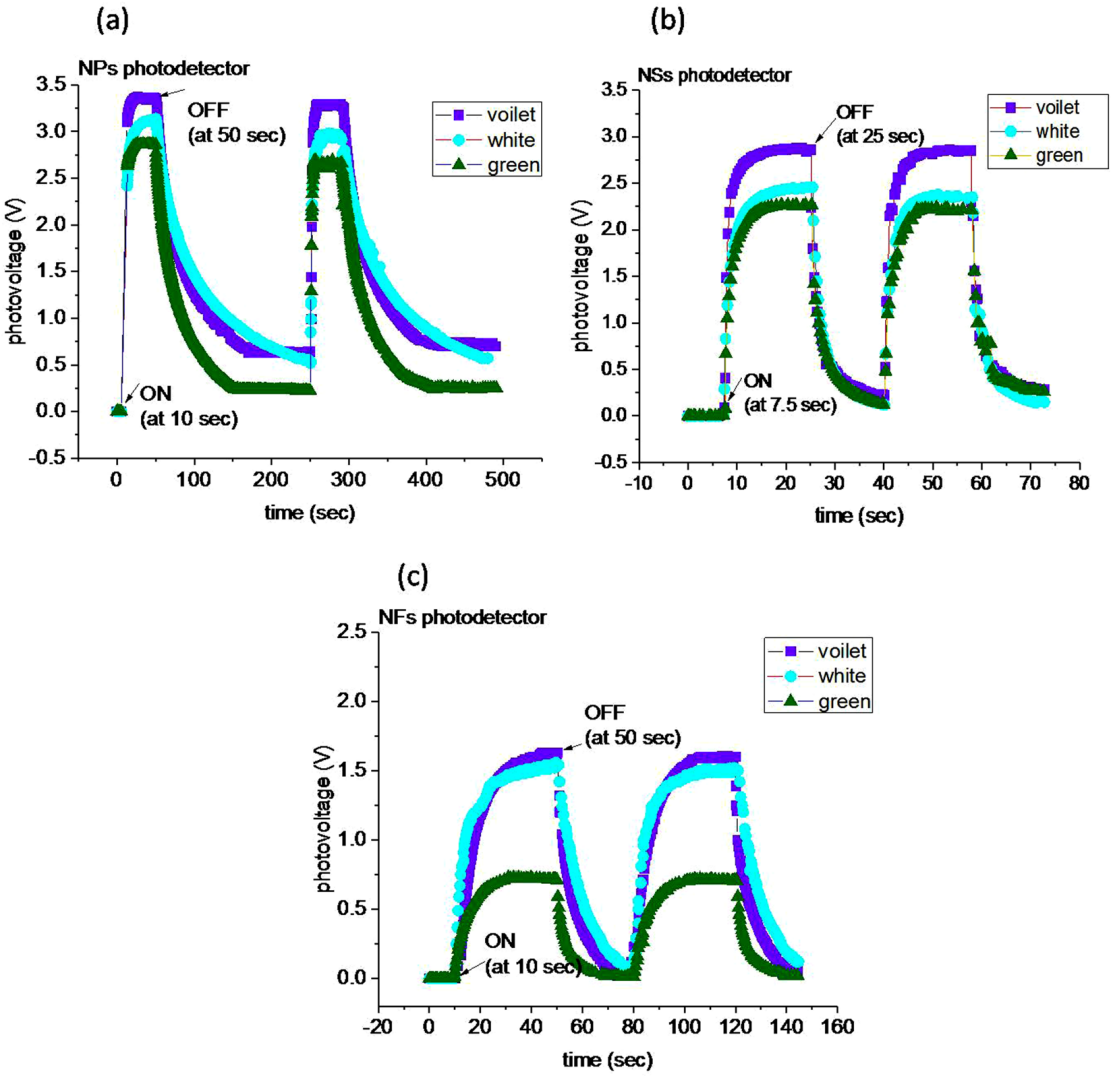
Comparison of photovoltage vs time curves of ZnO photodetectors thin films for UV, white and green illuminations. **(a)** NPs photodetector response showing high photo-generated voltage, **(b)** NSs photodetector shows fast response for both rise and fall time, and **(c)** NFs photodetector is slow for rise time and intermediate for fall time, also in this case the photo-generated voltage is smaller than that of NPs and NSs photodetectors.

Since for the green illumination, the photo-generated voltage is comparatively lower due to small number of generated charger carriers. As soon as the illumination is turned OFF, the produced smaller number of electrons will be captured by adsorbed O_2_/H_2_O at a faster rate than that for UV illumination^59^. Nevertheless, by UV illumination, electrons and holes are separated away in space that also increases their recombination life time^25^. However, the slow decay response in case of white illumination is unclear. When looking to the minimum value of dark voltage in the OFF state, there exists a shift in the dark voltage for each illumination. This may be ascribed to the presence of neutralized oxygen on the surface of ZnO-NPs. The neutralized oxygen residing on the surface would occupies a part of the surface, and thus will not allow the newly coming oxygen for electron capture, and thus resulting in a shift of photovoltage^60^. The observed shift in the dark voltage is in accordance with the energy leaves of illuminating radiation. The UV radiation, being a high energy, would neutralize more ions as compared with white and green illuminations, and therefore shows a larger shift in dark voltage (Fig. 8(a–c)).

Now let’s compare the photo-response of NPs, NSs and NFs photodetectors with respect to different illuminations (UV, white and green). The NSs and NPs photodetectors show similarity in their photovoltage rise time as 2 sec for UV, 4 sec for white and 5 sec for green; however, their ON state saturation voltages are different as shown in Fig. 8(a–c). NSs photodetector has smaller value of ON state saturation voltage than that of NPs photodetector. The NFs photodetector shows further smaller value of ON state saturation photovoltages as1.62 V for UV, 1.53 V for white and 0.72 V for green, whereas the photovoltage rise time is longer than that of NPs and NSs. The difference in the saturation photovoltages can be correlated with the different densities of illumination centers/defects in the nanostructures as detected by PL spectra (Fig. 4b). The observed PL intensities in the order, I_NF_ > I_NS_ > I_NP_ are representative of densities of illumination centers/defects in the respective nanostructure. These intensities/densities are adverse to the ON state saturation photovoltages. Indubitably, not all the peaks observed in PL do correspond to the generated photovoltage^61^, rather it indicates the possibility that a corresponding photovoltage may be generated upon illumination when any/all of the defect states participate in the photovoltage generation mechanism. The observed adverse effect of defect states over the ON state saturation voltage indicates that there should be capturing/scattering of photo-generated charge carriers during transportation^62,63^. In other words, the higher is defect density, lower is photo-generated voltage or vice versa. In whole of the process; however, unfortunately we could not obtained variation of defect states within a single morphology (either of NPs, NSs or NFs), as that could have been a further better examination of the effect of defects states variation over the ON state saturation voltage.

For decay/off state recovery time of photovoltages, it is known that the decay time depends mainly upon three factors; the available defect states (*V*_o_s and *Zn*_i_s in our case), surface area and adsorbate (O_2_ or H_2_O). The XPS results (Figs 5a and 6a) demonstrate that NPs possess smaller content of ${V}_{o}{\rm{s}}$ (21.8%) and smaller content of $Z{n}_{i}{\rm{s}}$ (as the main contribution being from hydroxide formation), whereas larger content of adsorbed O_2_/H_2_O (60.39%) along with a large surface area (20.5 m^2^/g) in comparison to that of NSs and NFs. The smaller percentage of *V*_*o*_s and *Zn*_*i*_s spreading over a larger surface area would initially promote fast chemisorption process of O_2_/H_2_O after turning OFF the illumination. The fast-initial decay can also be seen in NSs and NFs photodetectors, which latter on becomes slower and different in all the photodetectors. The fast decay, after turning OFF the illumination, indicates that initially the decay is essentially through a chemisorption process that is considered as a fast process. The surface states are then saturated by initial chemisorption process, and O_2_/H_2_O diffuse through the inter-crystallite deep into the surface, where they can find sites for adsorption; this corresponds to the slower decay^64^ of photovoltages. In NPs, the content of adsorbed O_2_/H_2_O (60.39%) over the 21.8% of ${V}_{o}{\rm{s}}$ and a smaller percentage of $Z{n}_{i}{\rm{s}}$ is larger, this shows that during the slower decay step, a large amount of O_2_/H_2_O, in comparison to the chemisorption, is physisorbed on the surface of NPs. Therefore, physisorption process is dominating in the case of NPs photodetector. Since the physisorption process involves rearrangement of adsorbate on the surface, so it will result in an increasing decay time of NPs photodetector (Fig. 8a). On the other hand, NSs photodetector, in comparison with NPs photodetector, has higher content of ${V}_{o}{\rm{s}}$ (47.7%) and $Z{n}_{i}{\rm{s}}$ (~13.3%), and smaller content of adsorbed (O_2_/H_2_O ~40.95%) along with smaller surface area (11.8 m^2^/g) would decay prominently through the chemisorption process due to the abundance of defect states to facilitate chemisorption process. Thus the larger content of *V*_*o*_s and *Zn*_*i*_s than the adsorbed O_2_/H_2_O leads to dominating fast chemisorption process that results in faster decay of NSs photodetector (Fig. 8b). Next, the NFs photodetector decay time shows totally different behavior, which is adverse to the trend obtained from NPs to NSs photodetectors. In this case, an increased decay time despite of higher content of ${V}_{o}{\rm{s}}$ (54.5%) and $Z{n}_{i}{\rm{s}}(28.6 \% )$, and smaller content of adsorbate (37.6%), surface area (10 m^2^/g) is observed. Here, the excess of defect states (*V*_*o*_s and *Zn*_*i*_s) appears to creates adverse effect on photoconductivity, similar to the reduction in the ON state saturation photovoltage (peak voltage in the Fig. 8(a,c)), and thus increases photovoltage decay time in NFs photodetector (Fig. 8c). The photovoltage rise time of NFs photodetector (15 sec for UV, 18 sec for white and 10 sec for green illuminations) is also longer than that of both the NSs and NPs photodetectors (Table 1). Here also, the deterministic factor appears to be the different content of *V*_*o*_s and *Zn*_*i*_s in nanostructures^64^; as the higher content of *V*_*o*_s and *Zn*_*i*_s in nanostructure photodetector results in longer photovoltage rise time. The NPs have lower percentage of *V*_*o*_s and *Zn*_*i*_s that would desorb rapidly and thus fast release of charge carriers upon illumination.

Form the above discussed observations, the UV and visible photo-response of thin film NSs is found better in comparison with NPs and NFs photodetectors (as compared in Table 1). Another important feature of NSs photodetector is its similarity in the rates of decay curves for both UV and visible illuminations despite of having different saturation voltages. The photo-response of the NSs photodetector in film architecture is even better than that of a sophisticated architectures consisting a single ZnO nanowire that performs only in UV illumination^24–26,57^.

## Conclusion

As a conclusion, a significant visible light photo-detection along with UV photodetection is achieved in pristine ZnO nanostructure based thin films. The defect states *V*_*o*_s and *Zn*_*i*_s along with morphology giving rise to the visible-light photo response are generated simply by tuning of solution method. The results of PL, XPS and photovoltages show that although the defect states *V*_*o*_s and *Zn*_*i*_s are responsible for exhibiting photo response in the visible-region of spectrum but at the same time their excess reduces the performance of photodetector as observed ~54% of ${V}_{o}{\rm{s}}$ and ~28% of $Z{n}_{i}{\rm{s}}$ in NFs photodetector, which is probable by scattering/capturing of charge carriers during their transportation. The ZnO-sheet based photodetector possessing moderate amount of ${V}_{o}{\rm{s}}( \sim \,47 \% )\,{\rm{and}}\,Z{n}_{i}s( \sim 13 \% )$ in comparison with ZnO-particle and ZnO-flower photodetectors, shows faster response to photovoltage growth time and decay time under the UV as well as visible-light illumination. The ZnO nanosheet based photodetector can be used as an efficient material for photodetector applications in broad-band spectral applications.

## Materials and Methods

### Synthesis of ZnO-NPs, ZnO-NSs, ZnO-NFs, and fabrication of photodetectors

The simple chemical route was tuned for the formation of surfactant free ZnO nanostructures. Detailed synthesis experiments listed in the Tables S1 and S2 of the supplementary information were performed with the variation of synthesis time, precursors, solvents and their molar ratio that resulted in the different ZnO morphologies. In the detailed experiments, a new approach was adopted to introduce ${V}_{o}{\rm{s}}$ and $Z{n}_{i}{\rm{s}}$ in the lattice of ZnO which enabled the ZnO nanostructures active for the visible light photo-detection. In the approach, an abrupt/fast mixing of precursor solution with alkali solution in the reaction chamber resulted in the formation of ${V}_{o}{\rm{s}}$ and $Z{n}_{i}{\rm{s}}$ as identified by XPS and PL studies. From the detailed synthesis experiments, we found that surfactant free ZnO-NSs and ZnO-NFs resulted only for specific conditions of precursor concentration (0.5 M), precursor’s molar ratio (1:1) and reaction time (4 hours). For NSs synthesis, 0.5 M solution of zinc chloride (ZnCl_2_) (purity 99.99%, Sigma-Aldrich, USA)) and 0.5 M solution of sodium hydroxide (NaOH) (purity 98%, Merck India Ltd.) were dissolved in ethyl alcohol (C_2_H_5_OH) separately. Then the prepared precursor solution of ZnCl_2_ was added abruptly in the solution of NaOH under vigorous stirring conditions at room temperature (~30 °C). After 4 hours of reaction time, the formed precipitate was collected, filtered, and washed with deionized water and C_2_H_5_OH to remove Cl^−^ and Na^+^ ions, which was finally dried at 60 °C. In the second set of experiments for the synthesis of NFs, all the conditions were kept same as that in case of NSs formation, the only solvent C_2_H_5_OH was replaced with deionized water. In this case, ZnO-NFs rather than NSs were obtained for the specific condition of precursor concentrations 0.5 M, precursor’s molar ratio 1:1 and reaction time 4 hours. The NPs were formed in both of the synthesis cases for precursor concentration 0.1 M, molar ratio of precursors 1:4 (NaOH: ZnCl_2_) and reaction time 15 min. In order to fabricate the photodetector of as synthesized ZnO nanostructures, they were dispersed in C_2_H_5_OH solutions and sonicated for 30 minutes. Then these dispersed nanostructures were spray coated on ultrasonically cleaned glass substrates to make uniform films of thickness about 4 µm. For spray coating, N_2_ was used as a carrier gas in the nozzle at spray rate of 1 ml/min. After drying, silver (Ag) interdigitated fingers were printed on the surface of films to make electrical contacts. The width of Ag interdigital electrodes was taken about 1 mm with fringe separation of 2 mm as shown in Figure S1.

### Characterizations of materials and photodetectors

The morphology of ZnO nanostructures was investigated by field-emission electron microscopy (FE-SEM, Hitachi S-4700, Tokyo, Japan), optical properties (absorbance and bandgap) were investigated by using UV-Vis spectrophotometer (Perkin-Elmer Lambda 750) and structural study was done by X-ray diffractometer (XRD) (Rigaku, radiation Cu kα, λ = 1.5406 Å). Photoluminescence (PL) (LS-55 Luminescence, Perkin Elmer, Germany), and X-ray photoelectron spectroscopy (XPS) were used to investigate defect states in the nanostructures. To estimate photovoltage in the visible region, a mercury lamp (power 100 watt) was used as a light source, whose intensity on the surface of photodetectors was adjusted 1 mW cm^−2^. The photovoltage of fabricated photo-detectors was recorded at room temperature using digital multimeter. In the photo-detection experiments, bias voltage of 5 V was applied. UV diode and optical filters with transmittance wavelength in the range 380~420 nm, 490~560 nm and λ > 420 nm (white light) were used to select different spectrum regions, respectively. The ‘ON’ and ‘OFF’ state of incident radiations were controlled by using a camera shutter. Schematic layout of the experimental set-up for the measurement of photo-generated voltage is shown in Figure S1.

## Electronic supplementary material

Supplementary Information
