# Supplementary material for: Visible and UV photo-detection in ZnO nanostructured thin films via simple tuning of solution method
Source: Sci Rep. 2017 Nov 8;7:15032. doi: 10.1038/s41598-017-15125-x (PMC5678174; doi:10.1038/s41598-017-15125-x)
Supplement: Supplementary file 1 — Supplementary Information [file 41598_2017_15125_MOESM1_ESM.pdf]

Visible and UV photo-detection in ZnO nanostructured thin films via simple tuning of solution method

Richa Khokhra[1], Bandna Bharti[1], Heung-No Lee[2*] and Rajesh Kumar[1,2*]

[1]Jaypee University of Information Technology, Waknaghat, Solan-173234, India
[2]Gwangju Institute of Science and Technology (GIST), 123 Cheomdangwagi-ro Buk-gu, Gwangju-61500, South Korea

The fast addition of reactants in the reaction chamber; a key parameter giving rise the distinct peaks in PL spectrums and high percentage of defect states in XPS studies, was optimized along with the variation of other reaction parameters such as; precursor concentration, precursor to alkali molar ratio and reaction time as mentioned in Tables S1 and S2. In total, about 20 experiments were performed in two sets, each consisting 10 experiments separately for solvent mediums; C_2H_5OH and H_2O, with respect to varying concentrations and molar ratio of precursor and alkali solutions. In all the experiments, mixing of reactant solutions was abrupt/fast (unlike the generally adopted method for ZnO nanostructure formation utilizing drop wise or slow addition of reactants)[1].

In the first step, using C_2H_5OH as the solvent medium and keeping the ratio as 1:1 of Zn precursor ($ZnCl_2$) and alkali solution (NaOH), the concentration was varied as mentioned in the first column of Table S1. The corresponding results obtained using the tabulated concentrations are shown inside the bracket. Initially, we used 0.1 M concentration for both the solutions and the obtained structure was unidentified morphology in aggregated form as shown in Figure S2a. For increased concentration to 0.2 M, some signatures of nucleation of sheets along with aggregated form was obtained as shown in Figure S2b. For 0.3 M concentration of reactants, slightly clear nanosheets structures emerging out of the aggregate appeared as shown in the Figure S2c. For 0.4 M concentration, the sheet-like structure becomes clearer as shown in Figure S2d; however, some part of aggregate is still present, and when the concentration was increased to 0.5 M, a clear nanosheets structure was obtained as shown in Figure S2e. For further higher concentration, 0.6 M, irregular structures were obtained which are not shown here. The result showed that the optimum concentration of reactants is 0.5 M.

Along with the molar ratio 1:1 of OH^- to Zn^{2+}, the concentration of these reactants also plays an important role in the formation of nanostructure. The $ZnCl_2$ concentration was kept

fixed (0.5M), whereas the concentration of NaOH was changed as; 0.125 M, 0.250 M, 0.50 M, 1.0 and 2.0 M as mentioned in second column of Table S1. The corresponding results are shown in Figures S3 a, b, c, d and e, respectively. For 0.125 M concentration of NaOH solution, the result shows formation of aggregated nanoparticles (Figure S3a), which for increased concentration of NaOH started to assemble into sheet type structures along with some nanoparticles on their surface as shown in Figure S3b. For higher concentration of NaOH as 0.5 M (equal to the concentration of Zn precursor as 0.5 M and becoming the ratio 1:1) there was formation of nanosheets alone (Figure S3c), and successively for further higher concentrations (such as 1.0 and 2.0 M of NaOH), the ZnO structures started to dissolve in the alkali medium, as the ZnO is an amphoteric compound which starts to dissolve in higher concentration of alkali solution, and therefore resulted in the hierarchical structure as shown in Figure S3(d and e). These results indicates that the molar ratio 1:1 is optimum ratio for the formation of distinguishable nanosheets of ZnO as the resultant of the reaction.

To estimate optimum time for perfect formation of nanosheet structures, samples were collected at 1, 2, 3 and 4 hours of time intervals of the reaction which was performed with concentration 0.5 M of NaOH and 0.5 M of $ZnCl_2$ (in the molar ratio 1:1). The perfect nanosheets samples were obtained for 4 hours of reaction time as shown in Figure S3c.

Similar to the first set of experiments conducted by using C_2H_5OH solvent medium, second set of experiments was performed using H_2O medium. Here alsothe molar ratio of OH^- : $Zn^{2+}$was kept 1:1, and concentration of Zn precursor and alkali solution was varied as 0.1, 0.2, 0.3, 0.4, 0.5 and 0.6 M as shown in first column of Table S2. For 0.1 M concentration of reactant ($ZnCl_2$ and NaOH) solutions, irregular microstructures were obtained as shown in Figure S4a, which for concentration 0.2 M resulted in aggregated sheets (Figure S4b). For higher concentration 0.3 M, flowers like of structures along with microstructures appeared in the resultant sample as shown in Figure S4c, and for further higher concentrations 0.4 M results are large amount of flower like structures along with some microstructures (Figure S4d). And in the last for 0.5 M concentration, a clear flower shaped nanostructured was obtained as shown in Figure S4e.

Again, in the second step the $ZnCl_2$ concentration was kept fixed (0.5M), whereas the concentration of NaOH was changed as 0.125 M, 0.250 M, 0.50 M, 1.0 and 2.0 M as shown in second column of Table S2. The corresponding FE-SEM results are shown in Figures S5a, b, c, d

and e, respectively. The NaOH concentration 0.125 M resulted in layered small sheet-like structures as shown in Figure S5a. For 0.25 M, it shows the formation of nanoparticles trying to arrange in a pattern (Figure S5b) and 0.5 M (reactants becoming in the ratio 1:1) resulted in flower type structure of ZnO as shown in Figure S5c. For further higher concentrations as 1.0 M and 2.0 M of NaOH, microstructures were obtained as shown in Figures S5d and e.

In this case too, a clearnanoflowers structure was formed in 4 hours of reaction time, with concentrations of reactants as 0.5 M of NaOH and 0.5 M of $ZnCl_2$ (resulting in ratio 1:1) as shown in Figure S4f.

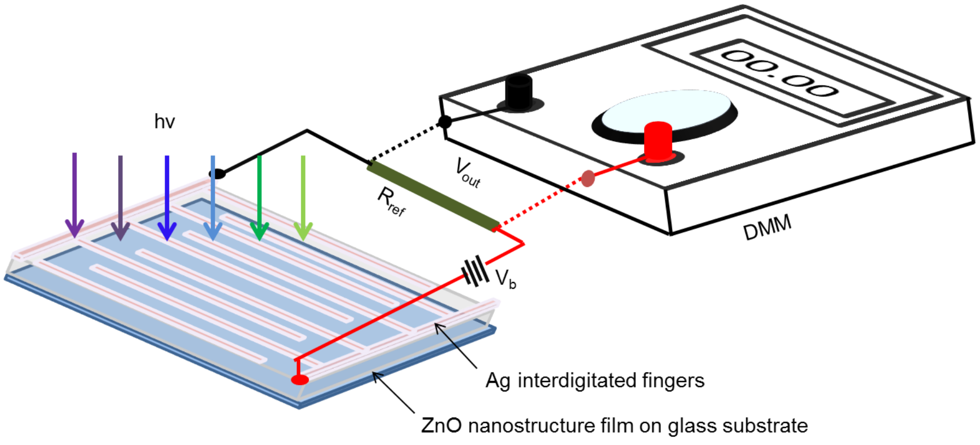

Figure S1 Schematic representation of photo-detection experiment. Bias voltage V_b= 5V and reference resistance R_{ref} = 4.7MΩ.

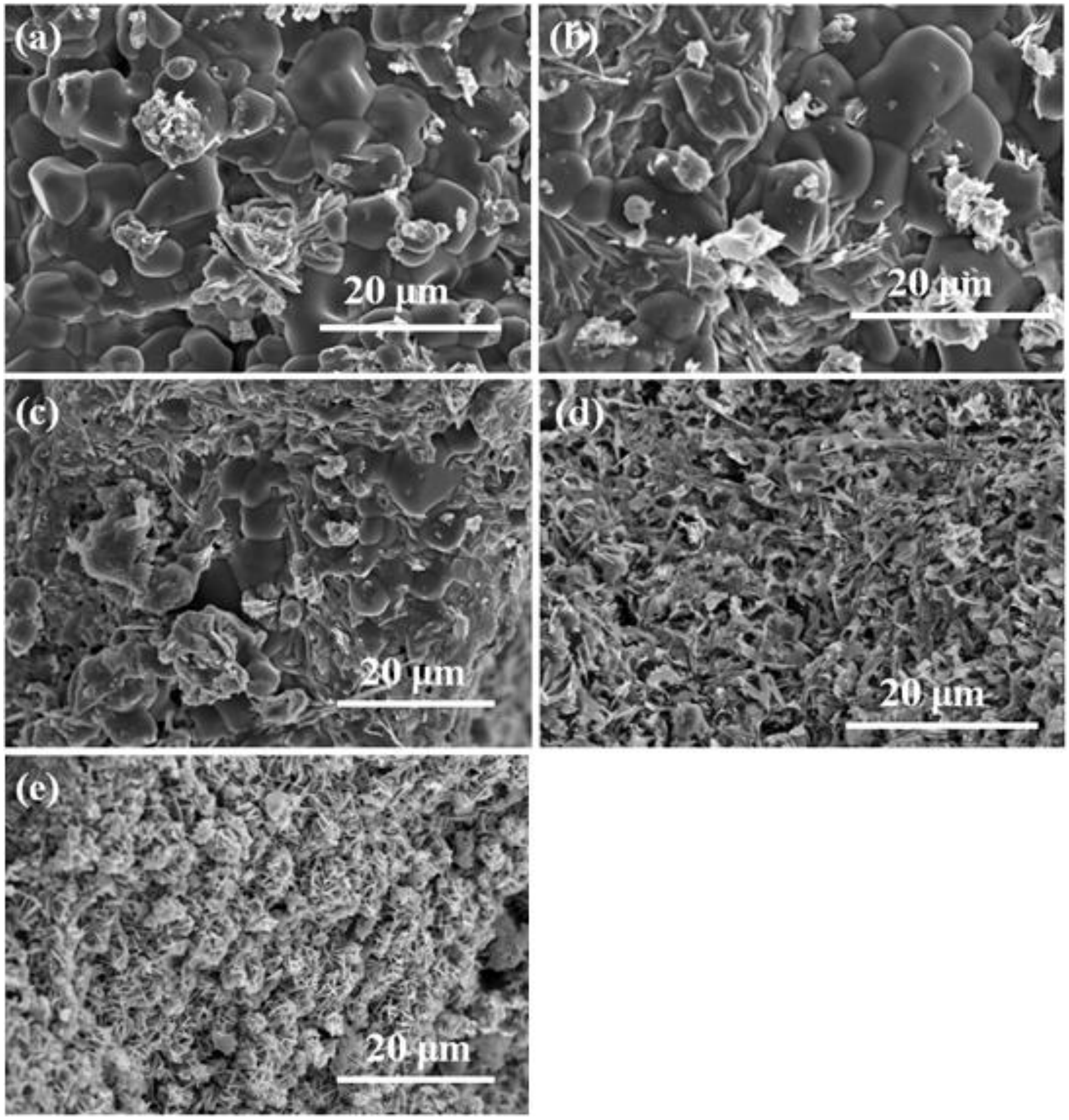

Figure S2FE-SEM images show variation in morphology by varying precursor and alkali solution concentration. (a) 0.1 M, **(b)** 0.2 M, **(c)** 0.3 M, **(d)** 0.4 M, **(e)** 0.5 M, keeping OH^-: Zn^{2+} ratio= 1:1. All the experiments were performed in C_2H_5OH medium.

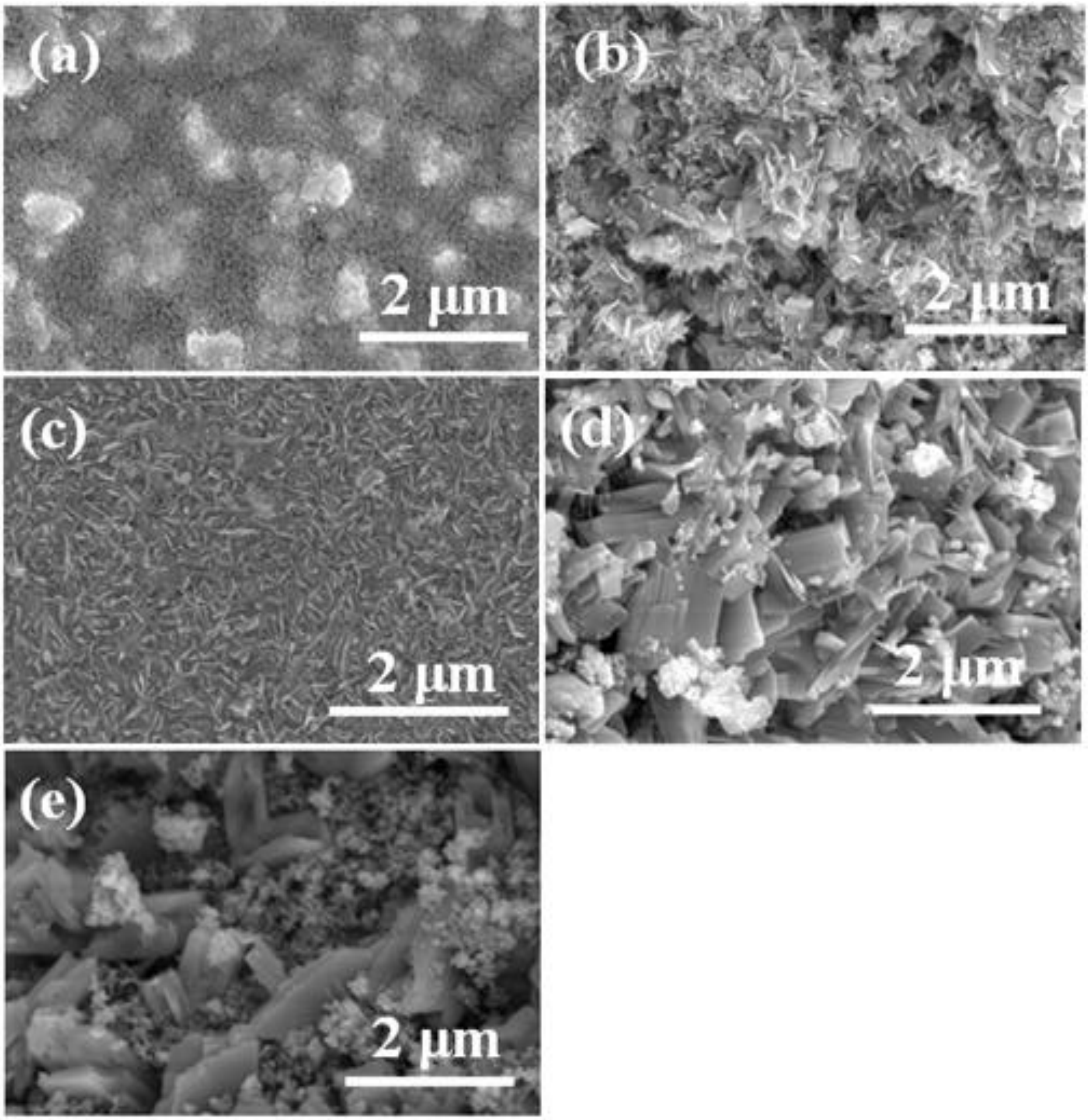

Figure S3 FE-SEM images show changes in morphology by variation of OH^- : Zn^{2+} ratio. (a) 1: 4,**(b)** 1: 2, **(c)** 1: 1, **(d)** 2: 1, and **(e)** 4: 1. The formation of NSs can be seen for 1:1 ratio of OH^- : Zn^{2+}.The experiments were performed in C_2H_5OH medium.Nanosheets obtained for 4 hours of reaction time are shown in (c).

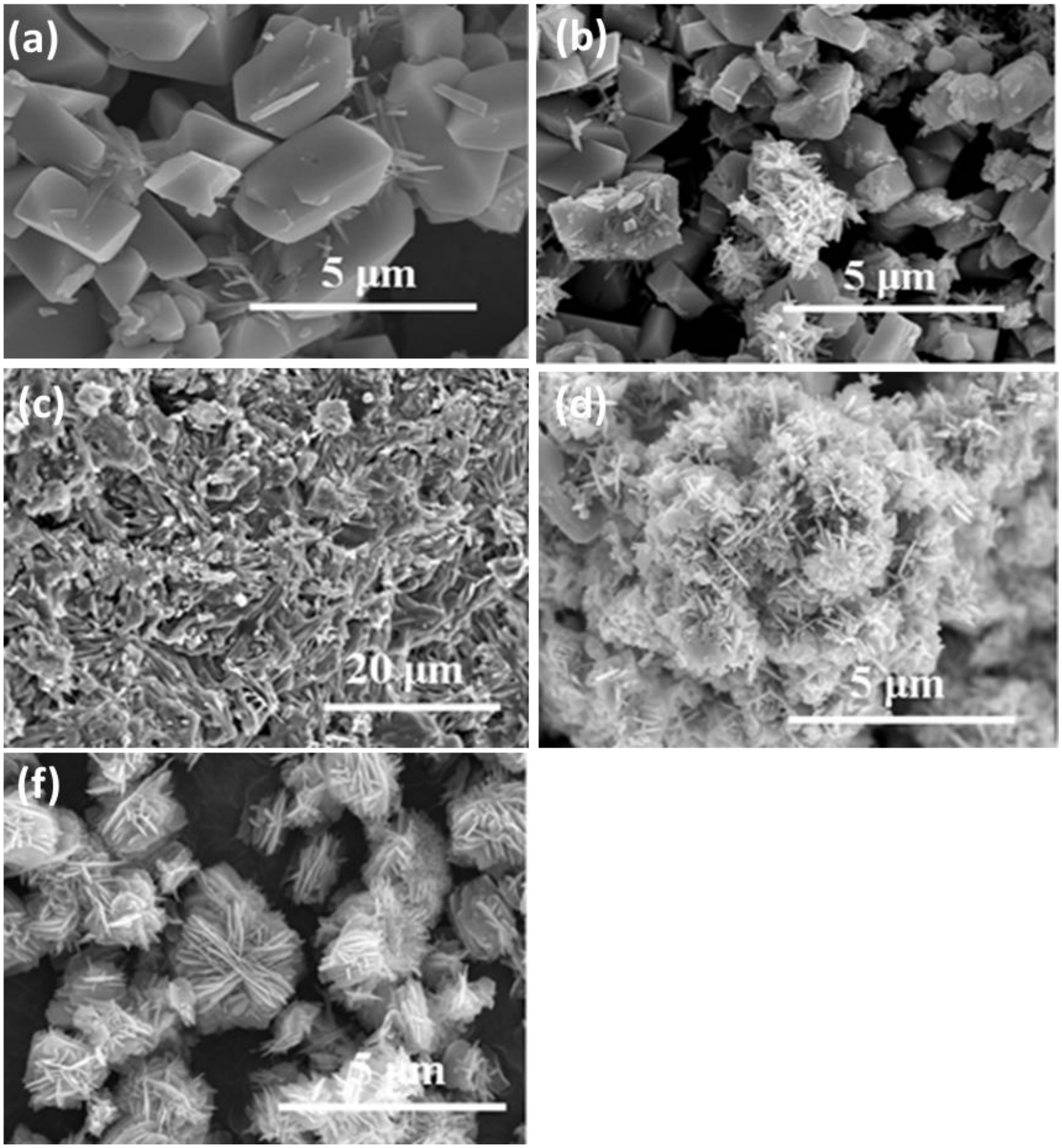

Figure S4 FE-SEM images show small change in the morphology with variation of concentration of precursor and alkali solutions simultaneously as **(a)** 0.1 M, **(b)** 0.2 M, **(c)** 0.3 M, **(d)** 0.4 M, **(e)** 0.5 M, keeping OH^-: Zn^{2+}=1: 1. The experiments were performed using H_2O medium. The nanoflowers formed in 4 hours of reaction time are shown in (f).

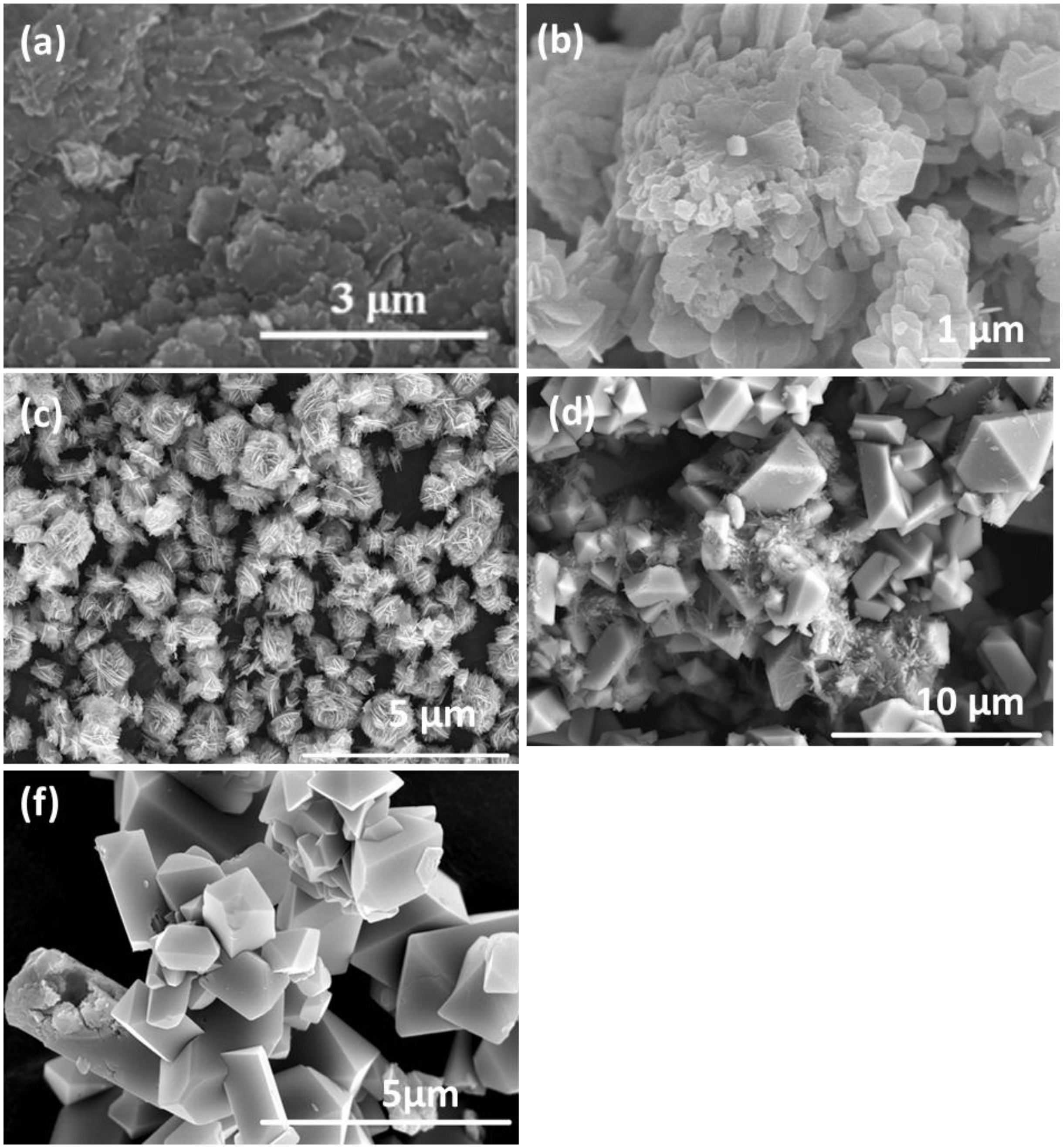

Figure S5FE-SEM images show change in the morphology by varying alkali to precursor solution ratio (OH^-: Zn^{2+}).(a) 1: 4,**(b)** 1: 2, **(c)** 1: 1, **(d)** 2: 1, and **(e)** 4: 1., respectively, in H_2O medium.

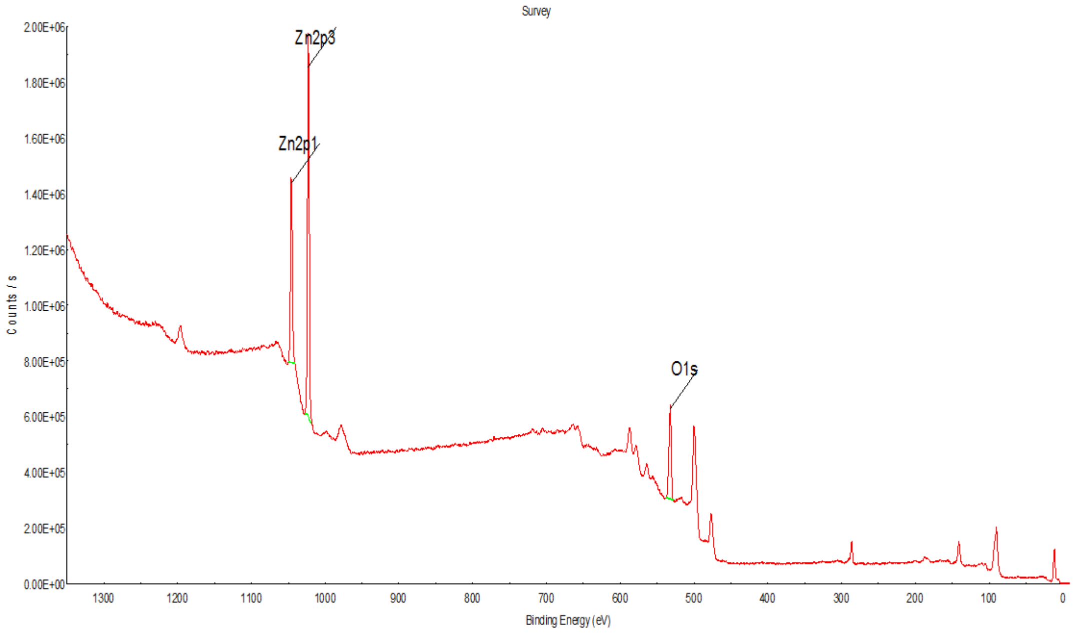

Figure S6 (a) XPS survey spectrum of NPs indicating presence of O1s and Zn2p peaks.

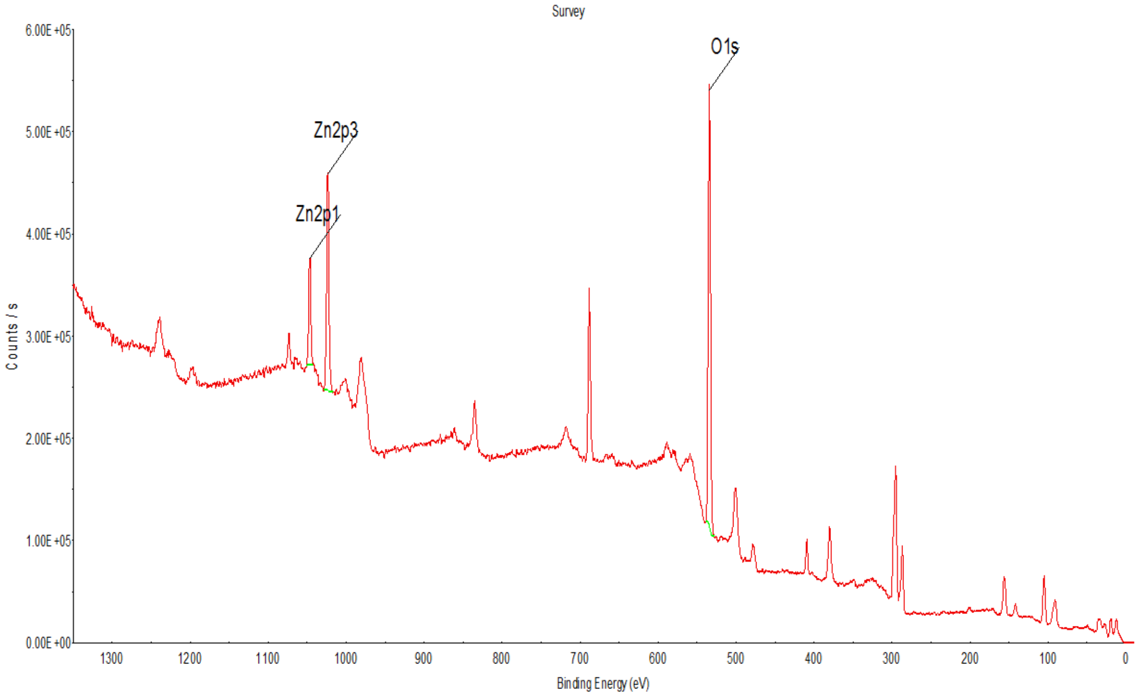

Figure S6 (b) XPS survey spectrum of NSs indicating presence of O1s and Zn2p peaks.

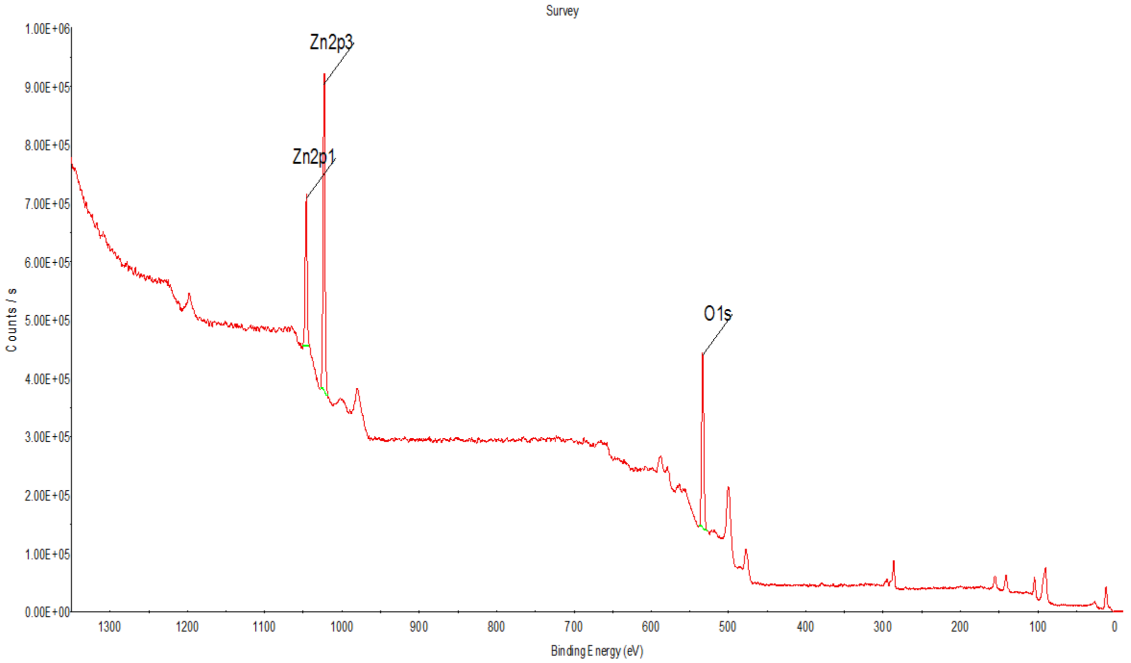

Figure S6 (c) XPS survey spectrum of NFs indicating presence of O1s and Zn2p peaks.

Table S1 detailed experiments for optimization of condition for synthesis of ZnO- NSs using C_2H_5OH solvent. The ZnO-NSs resulted only for specific conditions of precursor concentration (0.5M), precursor's molar ratio (1:1) and reaction time (4 hours). The corresponding output/results morphology is bracketed in the table.

**Variation in concentration Zn^{2+} and OH^-**	**Variation in ratio of OH^- and Zn^{2+}**	**Variation in reaction time (conc. of Zn^{2+} and OH^-= 0.5M, and OH^-:Zn^{2+} = 1:1)**
0.1M and 0.1 M (irregular microstructure)	1: 4 (nanoparticles)	15 minutes (nanoparticles)
0.2 M and 0.2 M (irregular microstructure)	1: 2 (appearance of nanosheets with nanoparticles)	1 hour (nanosheets with nanoparticles on their surface)
0.3 M and 0.3 M (appearance of nanosheets with microstructure)	**1: 1 (only nanosheets) conc. of both is 0.5M and 0.5 M**	2 hours (more nanosheets with some particles on surface)
0.4 M and 0.4 M (nanosheets in large amount as compared with microstructures)	2: 1 (nanosheets with nanoparticles on their surface)	**4 hours (only nanosheets structures)**
**0.5 M and 0.5 M (only nanosheets structures)**	4: 1 (nanosheets along with microstructures)	6 hours (only nanosheets structures)

Table S2 detailed experiments for optimization of condition for synthesis of ZnO- NFs using H_2O solvent. The ZnO-NFs resulted only for specific conditions of precursor concentration (0.5M), precursor's molar ratio (1:1) and reaction time (4 hours). The corresponding output is bracketed in the table.

**Variation in conc. of Zn^{2+} and OH^-**	**Variation in ratio of OH^- and Zn^{2+}**	**Variation in reaction time (conc. of Zn^{2+} and OH^-= 0.5M, and OH^-:Zn^{2+} = 1:1)**
0.1M<br><br>(irregular microstructure)	1: 4<br><br>(nanoparticles)	15 minutes<br><br>(aggregated nanoparticles)
0.2 M<br><br>(irregular microstructure)	1: 2<br><br>(appearance of flower-like structure with nanoparticles)	1 hour<br><br>(flower-like structure with nanoparticles on their surface)
0.3 M<br><br>(appearance of flower-like structure with microstructure)	**1: 1**<br><br>**(only flower-like structure)**<br>**Conc. of both are 0.5M**	2 hours<br><br>(more flower-like structure with some particles on surface)
0.4 M<br><br>(flower-like structure in large amount as compared with microstructures)	2: 1<br><br>(flower-like structure with nanoparticles on their surface)	**4 hours**<br><br>**(only flower-like structures)**
**0.5 M**<br><br>**(only flower-like structure)**	4: 1<br><br>(flower-like structure with microstructures)	6 hours<br><br>(only flower-like structure

References

1. Brintha, S. R. & Ajitha, M. Synthesis and characterization of ZnO nanoparticles via aqueous solution, sol-gel and hydrothermal methods. *IOSR J. Appl. Chem.* **8,** 66–72 (2015).
